# Spectral characterization of intraoperative renal perfusion using hyperspectral imaging and artificial intelligence

**DOI:** 10.1038/s41598-024-68280-3

**Published:** 2024-07-27

**Authors:** A. Studier-Fischer, M. Bressan, A.bin Qasim, B. Özdemir, J. Sellner, S. Seidlitz, C. M. Haney, L. Egen, M. Michel, M. Dietrich, G. A. Salg, F. Billmann, H. Nienhüser, T. Hackert, B. P. Müller, L. Maier-Hein, F. Nickel, K. F. Kowalewski

**Affiliations:** 1 Department of General, Visceral, and Transplantation Surgery, Heidelberg University Hospital, Heidelberg, Germany; 2 Department of Urology and Urosurgery, Medical Faculty of the University of Heidelberg, University Medical Center Mannheim, Mannheim, Germany; 3 Division of Intelligent Systems and Robotics in Urology (ISRU), German Cancer Research Center (DKFZ) Heidelberg, Heidelberg, Germany; 4 DKFZ Hector Cancer Institute at the University Medical Center Mannheim, Mannheim, Germany; 5 Division of Intelligent Medical Systems, German Cancer Research Center (DKFZ) Heidelberg, Heidelberg, Germany; 6 HIDSS4Health – Helmholtz Information and Data Science School for Health, Karlsruhe, Heidelberg, Germany; 7 National Center for Tumor Diseases (NCT) Heidelberg, a partnership between DKFZ and Heidelberg University Hospital, Heidelberg, Germany; 8 Faculty of Mathematics and Computer Science, Heidelberg University, Heidelberg, Germany; 9 Department of Anesthesiology, Heidelberg University Hospital, Heidelberg, Germany; 10 Department of General, Visceral, and Thoracic Surgery, University Medical Center Hamburg-Eppendorf, Hamburg, Germany; 11 Department of Digestive Surgery, University Digestive Healthcare Center, Basel, Switzerland

**Keywords:** Hyperspectral imaging, Renal perfusion, Renal malperfusion, Translational research, Porcine model, Machine learning, Surgery, Surgical data science, Preclinical research, Translational research

## Abstract

Accurate intraoperative assessment of organ perfusion is a pivotal determinant in preserving organ function e.g. during kidney surgery including partial nephrectomy or kidney transplantation. Hyperspectral imaging (HSI) has great potential to objectively describe and quantify this perfusion as opposed to conventional surrogate techniques such as ultrasound flowmeter, indocyanine green or the subjective eye of the surgeon. An established live porcine model under general anesthesia received median laparotomy and renal mobilization. Different scenarios that were measured using HSI were (1) complete, (2) gradual and (3) partial malperfusion. The differences in spectral reflectance as well as HSI oxygenation (StO_2_) between different perfusion states were compelling and as high as 56.9% with 70.3% (± 11.0%) for “physiological” vs. 13.4% (± 3.1%) for “venous congestion”. A machine learning (ML) algorithm was able to distinguish between these perfusion states with a balanced prediction accuracy of 97.8%. Data from this porcine study including 1300 recordings across 57 individuals was compared to a human dataset of 104 recordings across 17 individuals suggesting clinical transferability. Therefore, HSI is a highly promising tool for intraoperative microvascular evaluation of perfusion states with great advantages over existing surrogate techniques. Clinical trials are required to prove patient benefit.

## Introduction

Sufficient tissue oxygenation is vital for sustained physiological tissue function. Insufficient oxygenation or insufficient perfusion in general contributes to increased morbidity and mortality during various pathological conditions such as stroke and myocardial infarction, but also acute kidney injury.

Accurate intraoperative assessment of kidney perfusion stands as a pivotal determinant in ensuring optimal postoperative outcome and preserving renal function. The meticulous evaluation of kidney perfusion, characterized by the intricate interplay of vascular dynamics and tissue oxygenation, assumes paramount significance in guiding surgical interventions. The kidneys, being integral orchestrators of metabolic equilibrium and hemodynamic stability, necessitate a nuanced comprehension of their perfusion status during surgical procedures. By elucidating the fine balance between perfusion pressure, regional blood flow, and oxygen delivery, clinicians can deftly navigate the intricate landscape of surgical decision-making.

Despite its required robustness in function, the kidney is a delicate organ that is prone to ischemic injury and general perfusion distress. The balance of blood loss due to off-clamp vs. the ischemic injury due to on-clamp partial nephrectomy is a fine line regularly encountered by surgeons performing parenchyma-sparing kidney resections, which is the gold standard of current renal cancer treatment^1,2^. There are clinical trials presenting a multiple-fold increased risk of developing a severe chronic kidney disease during follow-up when undergoing on-clamp partial nephrectomy controlling arterial inflow as required for nephron-sparing surgery^3^. Similarly, obstruction of arterial inflow and venous outflow during kidney transplantation can lead to graft failure and loss of function when it is not recognized in a timely manner^4^.

A discerning evaluation of kidney tissue oxygenation therefore not only mitigates the immediate risk of ischemic injury, but also predicates the long-term renal functional prognosis, delineating the trajectory of postoperative recovery and long-term health. Objective measures for renal perfusion include magnetic resonance imaging (MRI), phase contrast MRI, cine phase contrast MRI, dynamic contrast-enhanced MRI, blood oxygen level dependent MRI, arterial spin labeling MRI, x-ray computed tomography, positron emission tomography, ultrasonic flowmetry, laser-Doppler flowmetry and ICG-fluorescence^5^. While only the latter three have proven feasible for intraoperative application, they still did not achieve the clinical breakthroughs proclaimed when initially introduced and can only be perceived as macrovascular or surrogate indicators. However, it would be desirable or even imperative to have the opportunity of an evaluation method on a microvascular level.

Hyperspectral imaging (HSI) is an advanced spectral imaging method that already experienced intraoperative application to urological and visceral transplant procedures^6,7^ and has great potential regarding renal perfusion evaluation. HSI allows for microvascular evaluation of the renal parenchyma capturing the actual determining factor and not just the surrogate parameters compared to other technologies. Furthermore, it could already be shown for other organ entities that histopathological cell physiology strongly correlates with HSI oxygenation results^8^. With these aspects in mind, HSI is the optimal choice for an exploratory analysis regarding applicability for objective perfusion evaluation in kidney surgery. However, the complexity and high dimensionality of HSI data renders manual analysis unavailing, yet it bears a well-suited opportunity for the application of Artificial intelligence (AI) and specifically Machine Learning (ML) all the more.

ML was first defined in the 1950s as “the field of study that gives computers the ability to learn without explicitly being programmed”^9^. It is usually a promising approach to deal with a problem when a governing equation is almost impractical to represent, since it is data-driven and can empirically approximate the unknown model^10,11^. Large data volume and subtle patterns not distinguishable by the human cognition—the very nature of HSI data—resemble the prime example for successful ML applications and led to the decision to augment analysis of this study using AI. This combination of HSI and ML has already proven feasible and lucrative with artificial neural networks, support vector machines and random forest techniques being intensively used for HSI analysis over the past years^12,13^. Recently published reviews entirely dedicated to ML with regards to medical HSI provide an overview over existing works and recommend on data handling and data normalization, wavelength selection, feature dimensionality reduction and the architecture of convolutional neural networks (CNNs)^14^. In the case of models for classification, most of them were improved by applying common CNNs like Resnet to extract spatial and spectral features in hyperspectral images, which was adhered to in the development of this study.

The specific research question of this manuscript is the degree and nature of spectral differences of different renal perfusion states aiming to provide a first in-depth understanding of the respective spectral characteristics during kidney surgery.

## Results

### Complete malperfusion of the kidney by clamping renal arteries and veins in animals

**Figure 1:**
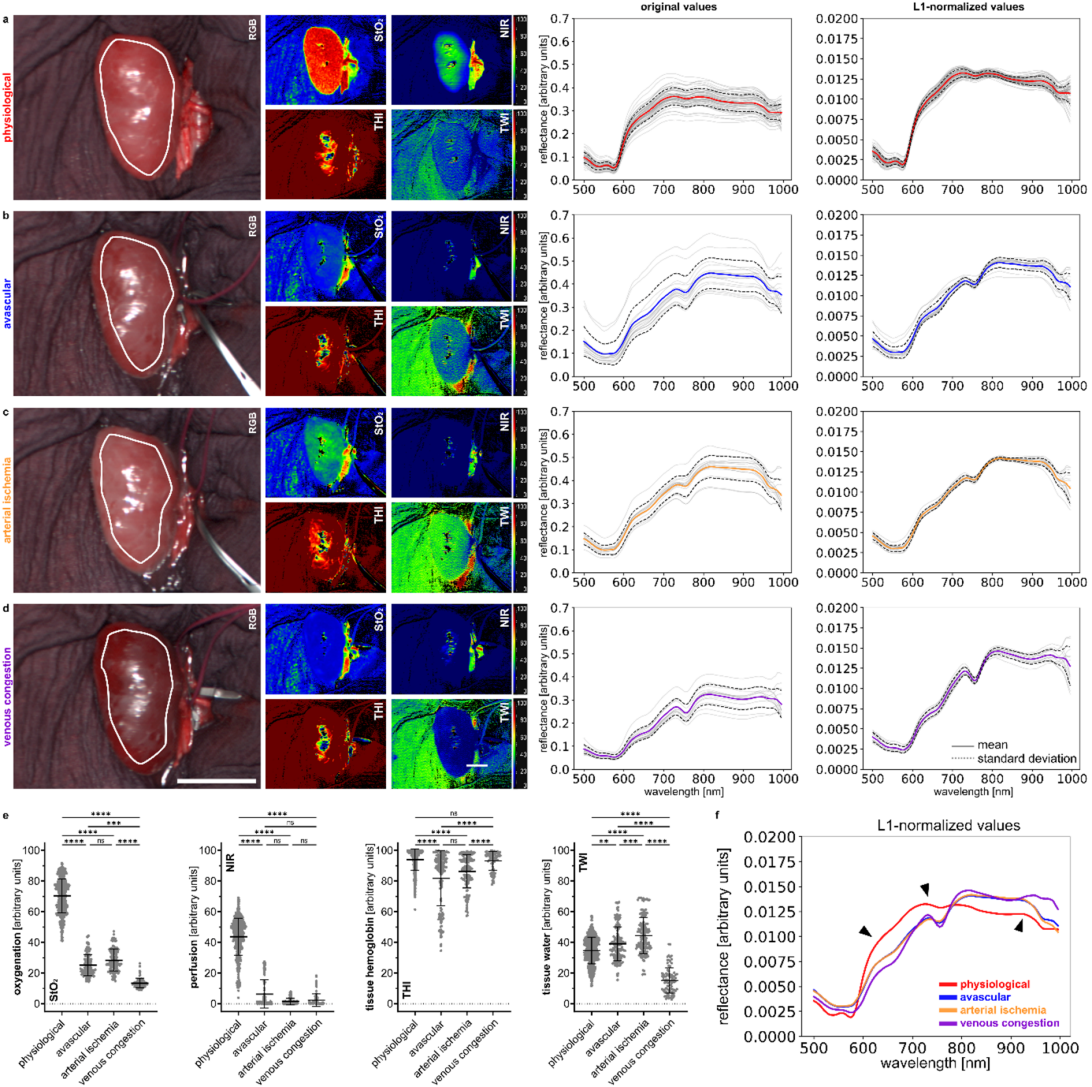
Complete malperfusion of the kidney by clamping renal arteries and veins. HSI Color-Index pictures and respective spectra for porcine kidney. (**a**) physiological (I = 54, n = 757). (**b**) avascular (I = 17, n = 162). **c**, arterial ischemia (I = 14, n = 156). (**d**) venous congestion (I = 15, n = 91). (**e**) quantification of HSI index values for StO_2_, NIR, THI and TWI. (**f**) comparison of L1-normalized reflectance spectra. White scale bar equals 5 cm.

There are four basic perfusion states imaginable for kidneys, i.e. (1) “physiological” perfusion, (2) “avascular” kidney (combined inhibition of arterial inflow and venous outflow), (3) “arterial ischemia” (selective inhibition of arterial inflow) and (4) “venous congestion” (selective inhibition of venous outflow). The spectral reflectances of these four perfusion situations are characteristic and did show relevant differences regarding their spectral curves (Fig. 1; Supplement Fig. 1). Yet “avascular” and “arterial ischemia” had great spectral similarity (Fig. 1f) and will therefore be collectively referred to as “combined compromised inflow” in some of the subsequent analyses. Relevant differences could also be seen considering corresponding HSI index values. While the “physiological” group had the highest values with 70.3% (± 11.0%) for StO_2_ and 43.7% (± 12.2%) for NIR, the “venous congestion” group achieved the lowest with 13.4% (± 3.1%) and 2.3% (± 4.1%). The “avascular” group presented with 25.2% (± 6.8%) for StO_2_ and 6.3% (± 9.2%) for NIR, while the “arterial ischemia” group presented with 28.4% (± 7.2%) and 1.4% (± 2.1%).

### Spectral signatures of different complete malperfusion states in animals form clusters when applied to multidimensionality reduction tools and can be differentiated by machine learning algorithms

**Figure 2:**
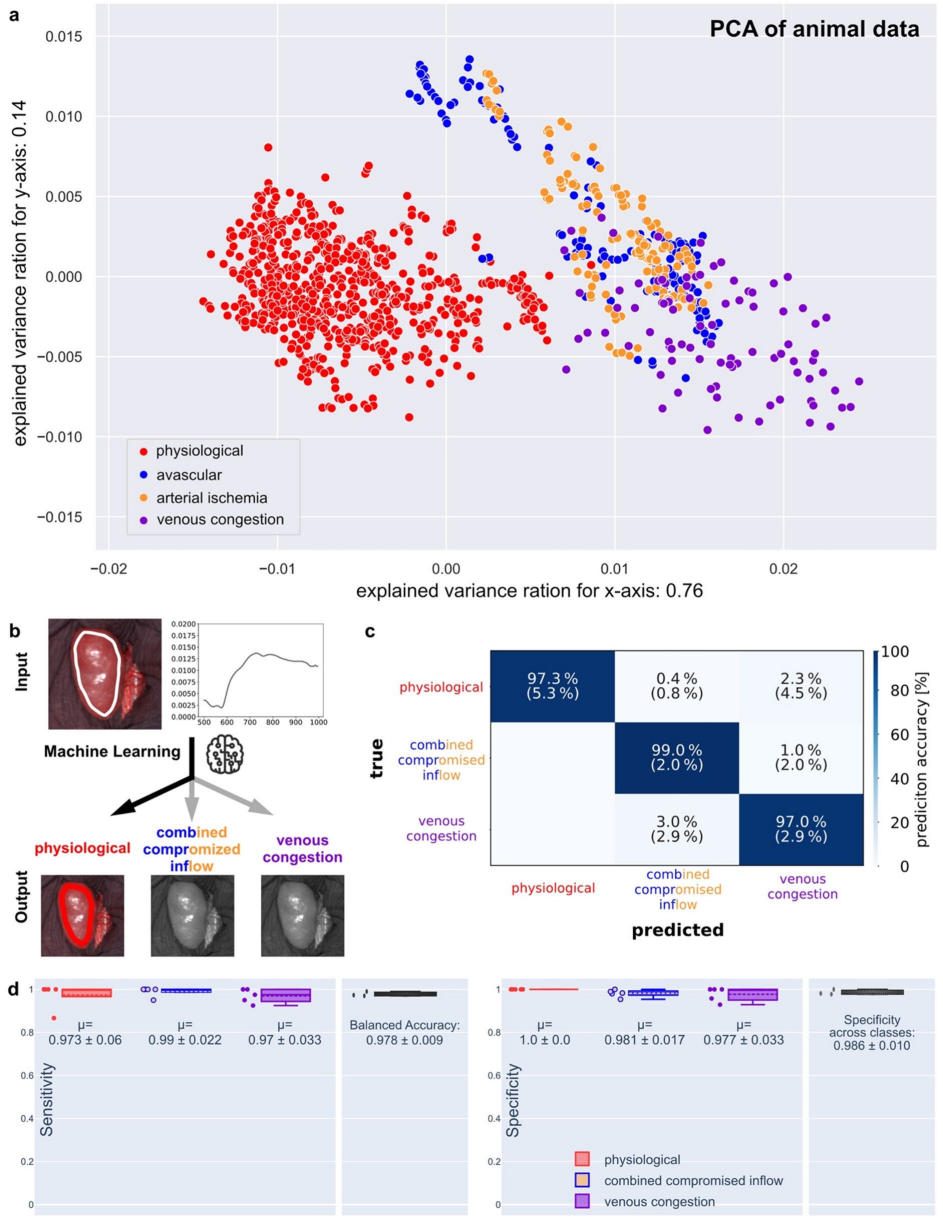
Comparison of data of complete renal malperfusion. (**a**) principal component analysis (PCA). (**b**) visualization of the machine learning classification process using “physiological” as an example. (**c**) contingency table of prediction accuracy. (**d**) quantification of sensitivity and specificity on the test set.

As HSI data is multidimensional, its primary information content cannot be comprehended by the human mind. Multidimensionality reduction tools such as principal component analysis (PCA) are able to condense contained information into tangible visualizations (Fig. 2). It can be seen that the four previously mentioned basic perfusion states form clusters that are mainly isolated such as for “physiological” and “venous congestion” or mainly overlap such as for “avascular” and “arterial ischemia” with an overall explained variance of around 90%. This confirms what was already apparent from the spectral comparison in Fig. 1b, i.e. that spectral similarity between the two latter groups is significantly high. However, the surgeon does not require a ML-based classification of all four. In analogy to the established paradigm “treat first what kills first” in emergency medicine, intraoperatively, a surgeon would in general first address arterial compromise before solving venous outflow issues. Consequently, for a ML-model with the aspiration of clinical relevance, it makes sense to merge the classes “avascular” and “arterial ischemia” when its prediction accuracy can hereby be increased. The surgeon would in both cases first address the arterial issue and only then reevaluate the organ to possibly solve another underlying venous compromise. This merger that was motivated from the comprehension of surgical strategy allowed for an impressive prediction accuracy of above 97% rendering the application of this diagnostic tool reliable and beneficial.

AI is well-suited in situations of complex and multi-dimensional data exceeding the human cognitive capacity. Therefore, a ML algorithm was trained and evaluated to separate the three groups “physiological”, “combined compromised inflow” and “venous congestion” (Fig. 2b). Our ML framework achieves a balanced prediction accuracy of 97.8% (Fig. 2c) and high levels of sensitivity as well as specificity (Fig. 2d) on separate hold-out test datasets. The prediction of an image is computationally efficient since the inference time for an image takes only about three milliseconds on a GeForce RTX™ 4090 (Nvidia Corporation, Santa Clara, USA).“

### Complete malperfusion of the kidney in animals causes characteristic changes in the reflectance spectra over time

When reversibly restricting organ perfusion through vascular clamping, organ physiology changes over time. Similarly, the new spectral state does not stabilize immediately. Instead, there is a transition into the new spectral state. Figure 3 visualizes this transition into the malperfused state. Supplement Figs. 2–14 elaborate on this and also depict the reverse process of reperfusion.

**Figure 3:**
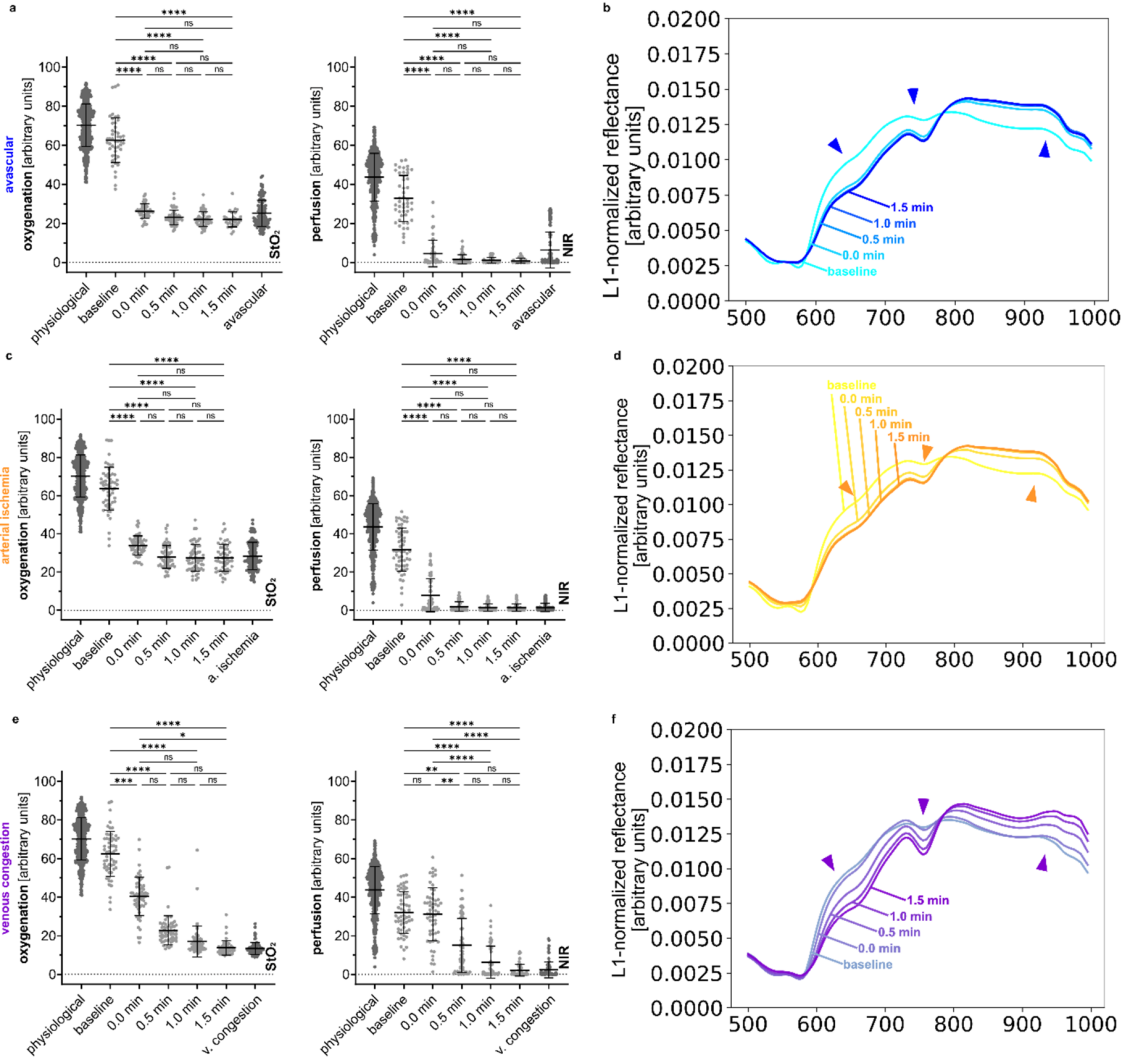
Spectral changes over time in different states of complete renal malperfusion. Quantification of HSI index values (StO_2_ and NIR) with reference boxplots from the baseline groups and comparison of multiple L1-normalized reflectances according to changes over time. (**a**, **b**), avascular. (**c**, **d**), arterial ischemia. (**e**, **f**), venous congestion.

It was observed that “avascular” and “arterial ischemia” are subject to a much more imminent and saltational transition as the spectral steady state is almost already reached directly at clamping (0.0 min). In contrast, the transition for “venous congestion” is more gradual and the steady state is reached only after 1.5 min. Therefore, measurements used for the baseline recordings from Fig. 1 were only used when recorded in an unequivocal steady state of longer than 4 min after clamping.

### Gradual malperfusion of the kidney by gradually clamping renal arteries and veins in animals

**Figure 4:**
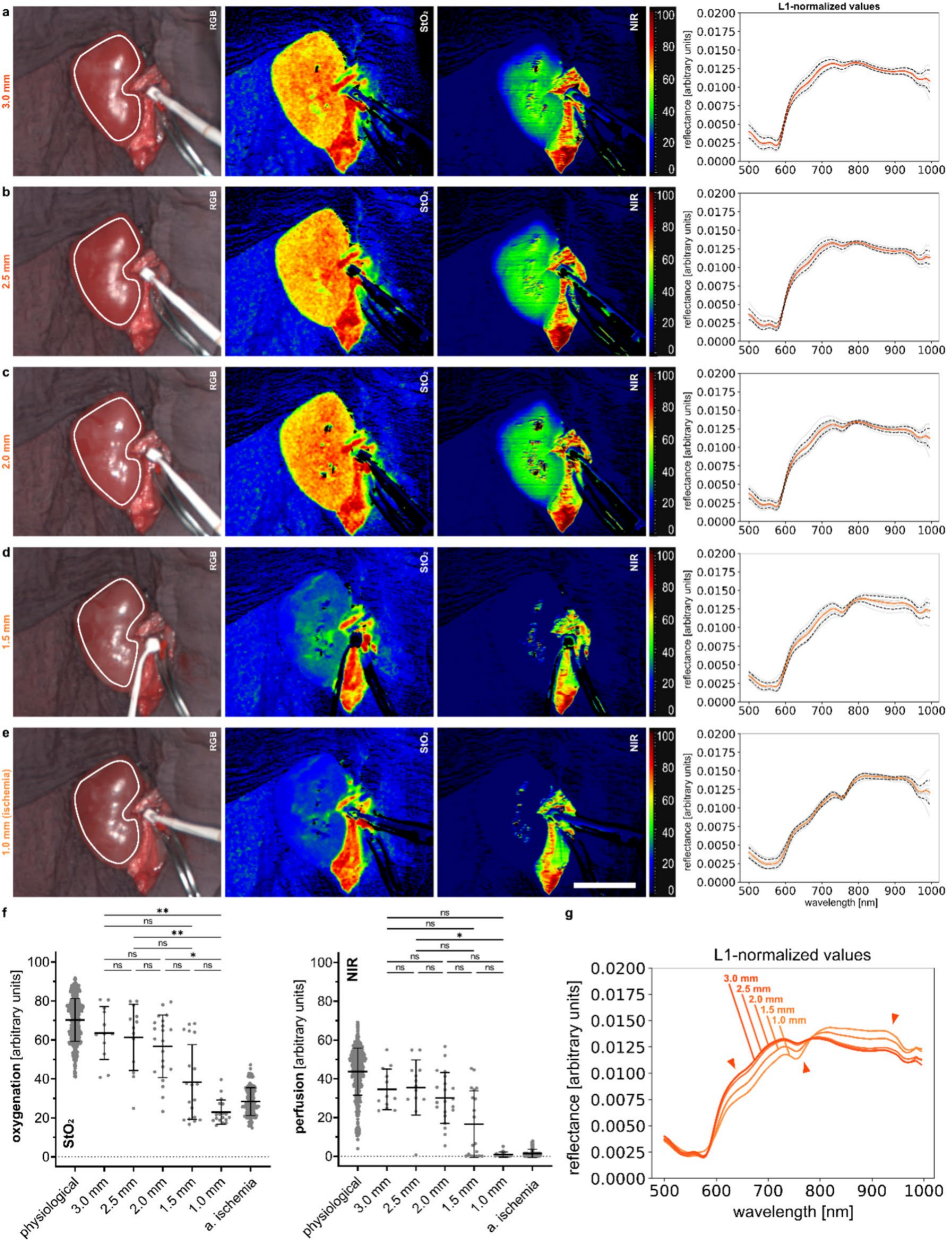
Gradual clamping of the renal arteries with various gap sizes. HSI color index pictures and respective spectra for porcine kidney. (**a**) gradually ischemic kidney with a 3.0 mm gap on the artery (I = 8, n = 12). (**b**) gradually ischemic kidney with a 2.5 mm gap on the artery (I = 8, n = 13). (**c**) gradually ischemic kidney with a 2.0 mm gap on the artery (I = 11, n = 21). (**d**) gradually ischemic kidney with a 1.5 mm gap on the artery (I = 9, n = 19). (**e**) gradually ischemic kidney with a 1.0 mm gap on the artery (I = 11, n = 18). (**f**) quantification of HSI index values for StO_2_ and NIR. (**g**) overlay of multiple L1-normalized reflectances according to the different gap sizes. White scale bar equals 5 cm.

More often than not, renal malperfusion is not due to complete, but to gradual inhibition of perfusion e.g. through anastomotic stenosis or strictures. In order to investigate this situation, renal arteries and veins were gradually occluded as indicated in Fig. 10c. Spectral changes for gradual clamping of the renal arteries could be observed with the most relevant changes between the transition from 2 mm to 1.5 mm of standardized stricture diameter simulation (Fig. 4 and Supplement Fig. 15). A first major spectral shift towards spectral properties of deoxygenated hemoglobin patterns was visible and StO_2_ and NIR values dropped from 56.7% (± 16.1%) and 30.1% (± 13.1%) to 38.4% (± 19.2%) and 16.6% (± 17.2%).

**Figure 5:**
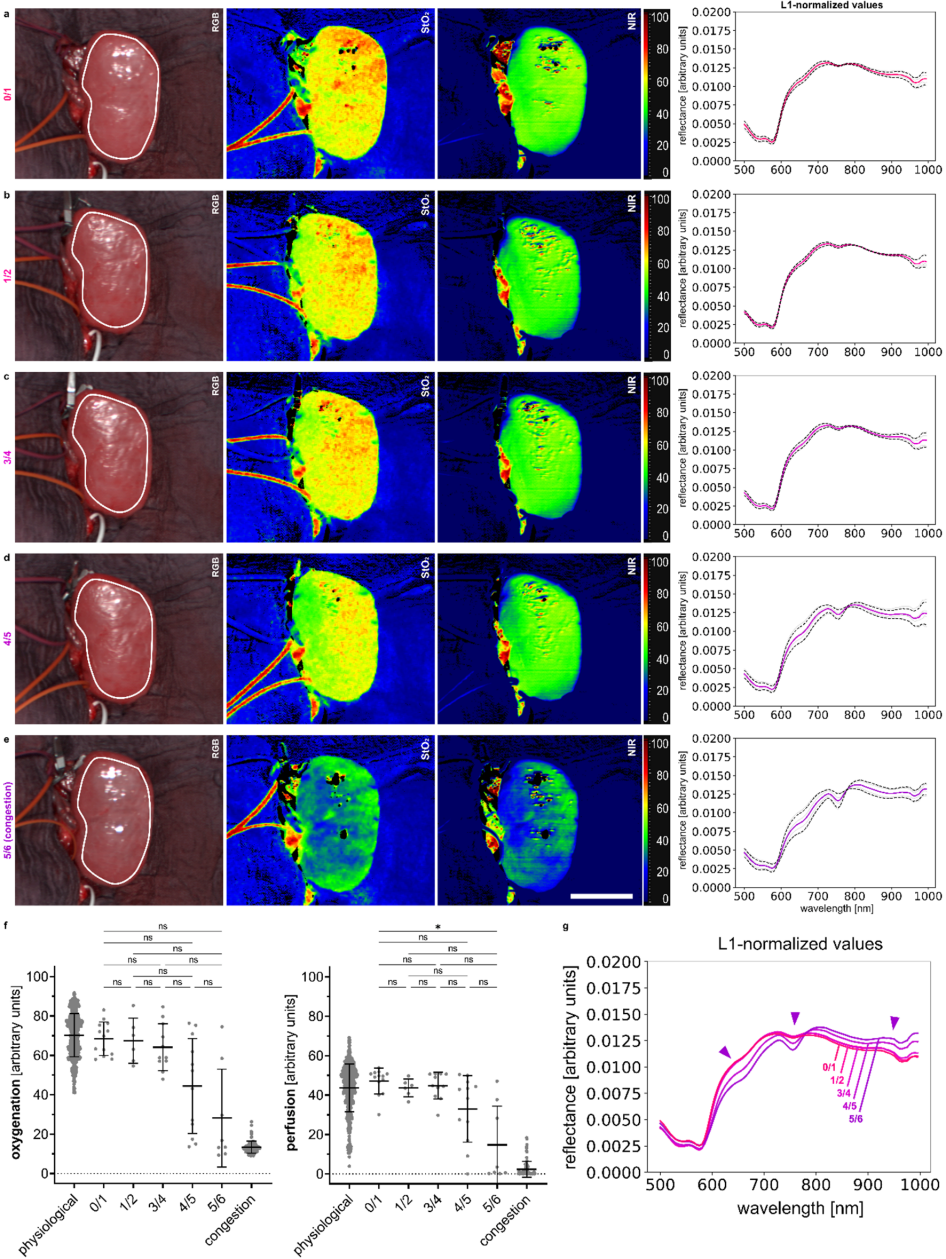
Gradual clamping of the renal veins with different steps of occlusion. HSI color index pictures and respective spectra for porcine kidney. (**a**) kidney with half occluded vein (I = 3, n = 6). (**b**) kidney with three-quarters occluded vein (I = 4, n = 11). (**c**) kidney with four-fiths occluded vein (I = 4, n = 12). (**d**) kidney with five-sixths occluded vein (I = 4, n = 8). (**e**) completely reperfused kidney after the process of gradual stasis (I = 4, n = 13). (**f**) quantification of HSI index values for StO_2_, NIR and TWI. **g**, overlay of multiple L1-normalized reflectances according to the different occlusion steps. White scale bar equals 5 cm.

Spectral changes for gradual clamping of the renal veins could also be observed with the most relevant changes between the transition from 3/4 to 4/5 occlusion of flattened vein diameter (Fig. 5 and Supplement Fig. 16). A first major spectral shift towards spectral properties of deoxygenated hemoglobin patterns was visible and StO_2_ and NIR values dropped from 64.2% (± 11.9%) and 44.8% (± 6.8%) to 44.4% (± 24.1%) and 33.0% (± 16.9%).

### Partial malperfusion of the kidney by selectively clamping pole arteries in animals

**Figure 6:**
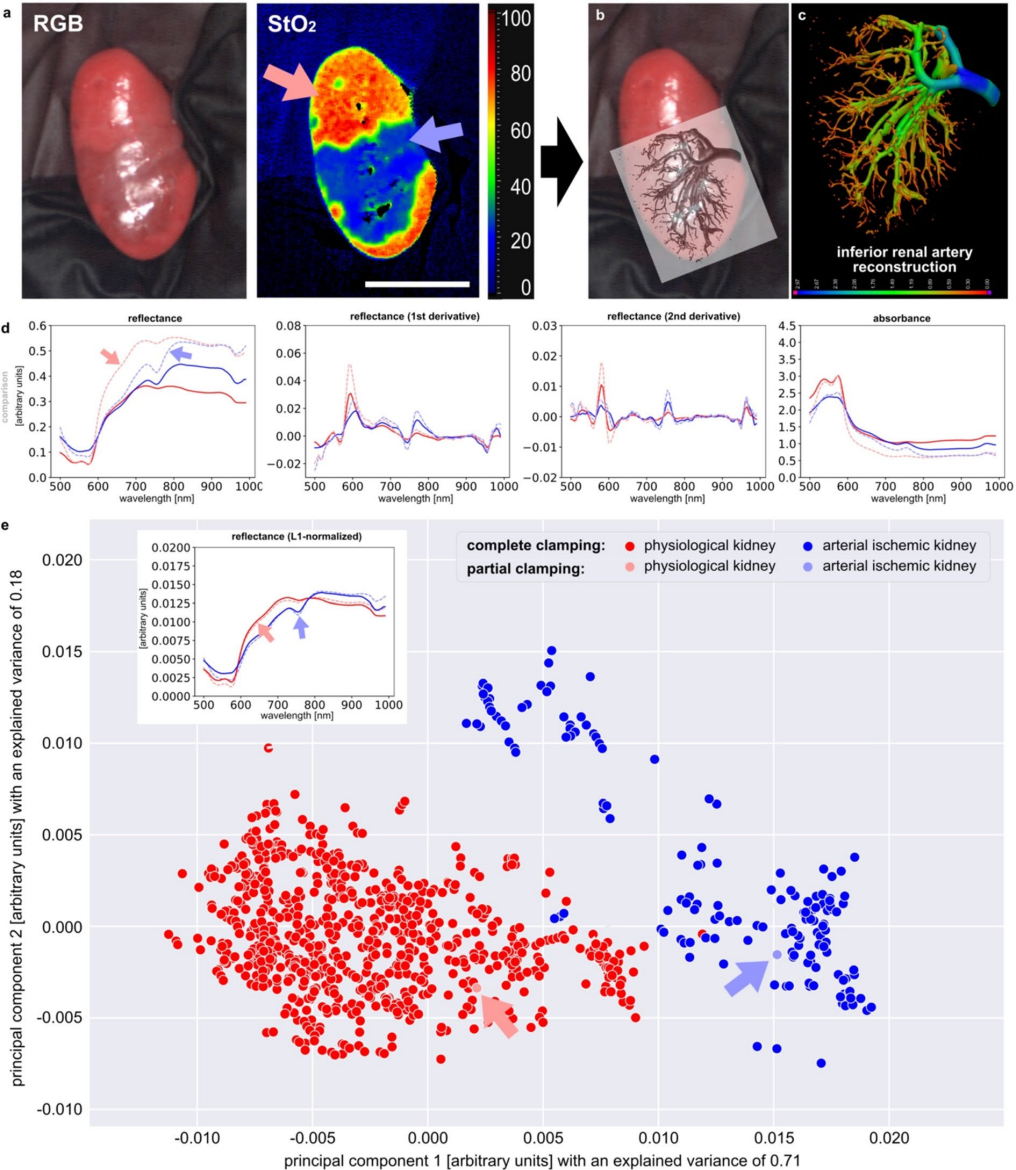
Partial malperfusion of the kidney by selectively clamping pole arteries in pigs. (**a**) example of porcine kidney with inferior renal artery malperfusion. (**b**, **c**) 3D-reconstruction of inferior renal artery from a vascular corrosion cast. (**d**) comparison to spectral data from complete malperfusion. (**e**) PCA combining exemplary data from complete and partial malperfusion. Arrows indicate the spectral measurement points from the recording in **a** relative to the already existing PCA for complete malperfusion.

The third and final situation encountered regularly in clinical scenarios besides (1) general malperfusion and (2) gradual malperfusion is (3) partial arterial malperfusion of the kidney. By inducing partial malperfusion with a vascular clamp to the inferior renal artery in the porcine model, the spectral reflectance signals of partial malperfusion could be extracted with high validity (Fig. 6). It became apparent that spectral signatures for partial and global arterial malperfusion are the same (Fig. 6d) and that the spectral measurements of partial arterial malperfusion associate to the existing PCA clusters measured for general malperfusion (Fig. 6e). Partial clamping results in a relatively sharply defined ischemic area of the renal parenchyma as seen in the StO_2_ images in Fig. 6a. In order to provide a pathomechanistic explanation, digital volume tomography (DVT) of the renal vascular corrosion casting was performed and the inferior renal arterial system was reconstructed and superimposed onto the HSI measurement (Fig. 6b,c, Supplement Fig. 17). It therefore became apparent that exactly the area dropping in StO_2_ saturation was also the area perfused by the inferior renal artery, confirming intuition and validating HSI StO_2_ measurements at the same time.

### Data from human samples indicate transferability

**Figure 7:**
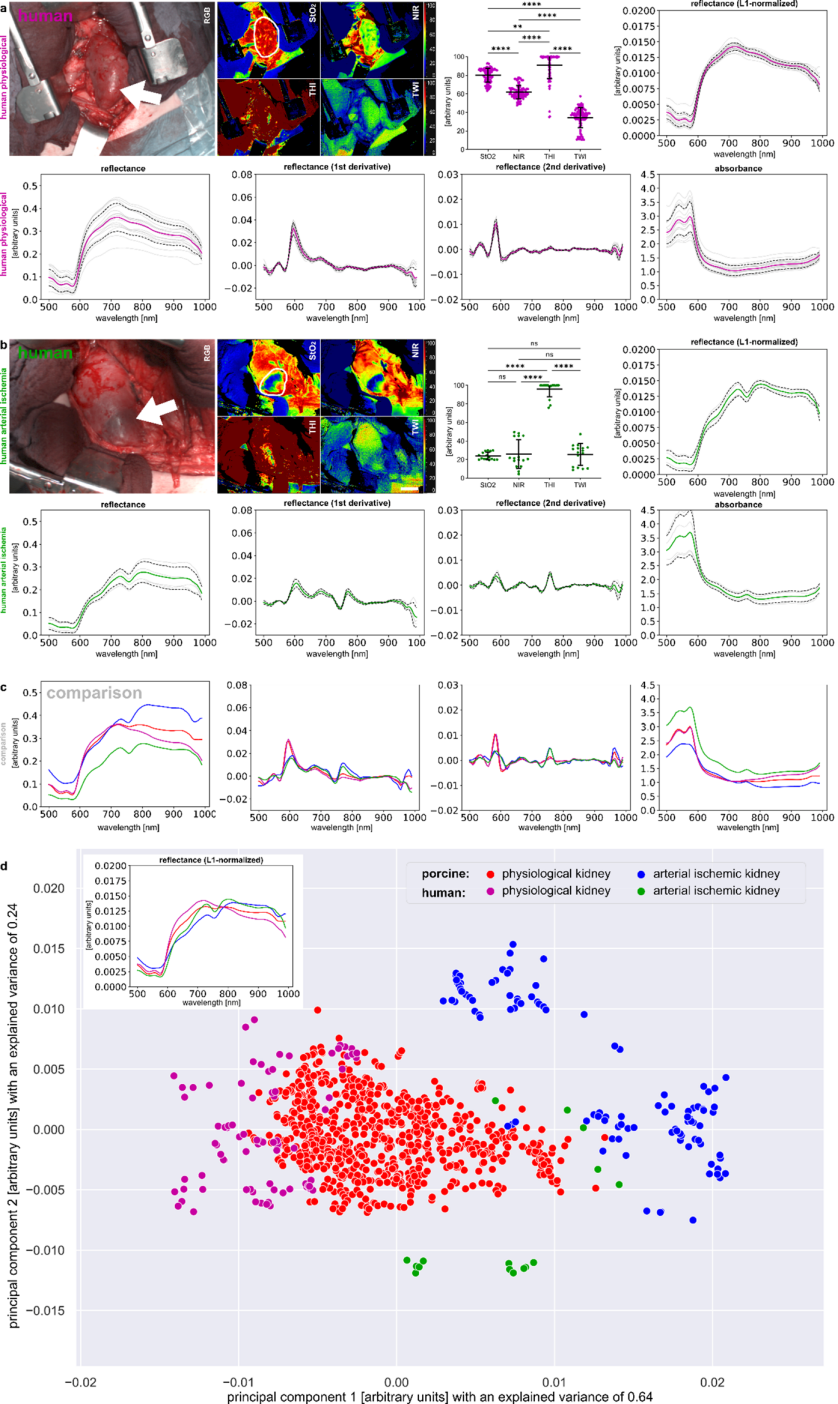
Human kidney data. (**a**) physiological human kidney (I = 17; n = 88). (**b**) human kidney with arterial ischemia (I = 4; n = 16). (**c**) comparison to porcine data. (**d**) PCA.

Data from this porcine study was compared to human data from the SPACE trial^15^ to foster the process of transferability (Fig. 7). Patient data was recorded from patients undergoing kidney transplantation. Recordings for human physiological renal perfusion were obtained from regular kidneys. Recordings for human arterial renal malperfusion were obtained from regions that depicted clear StO_2_ demarcation because vascular anastomosis of an inferior renal artery was deemed negligible and not performed during transplantation. In the inter-species comparison of L1-normalized spectra in Fig. 7d, it could be seen that although slight differences exist, the overall curve shape of measured reflectance is highly comparable between the two species for the respective physiological and arterial ischemic organ states. The PCA with an explained variance of 86% indicates partial overlap between the clusters especially for the two physiological groups.

### Arterial flow measured with flowmeter correlates with oxygenation and blood pressure

**Figure 8:**
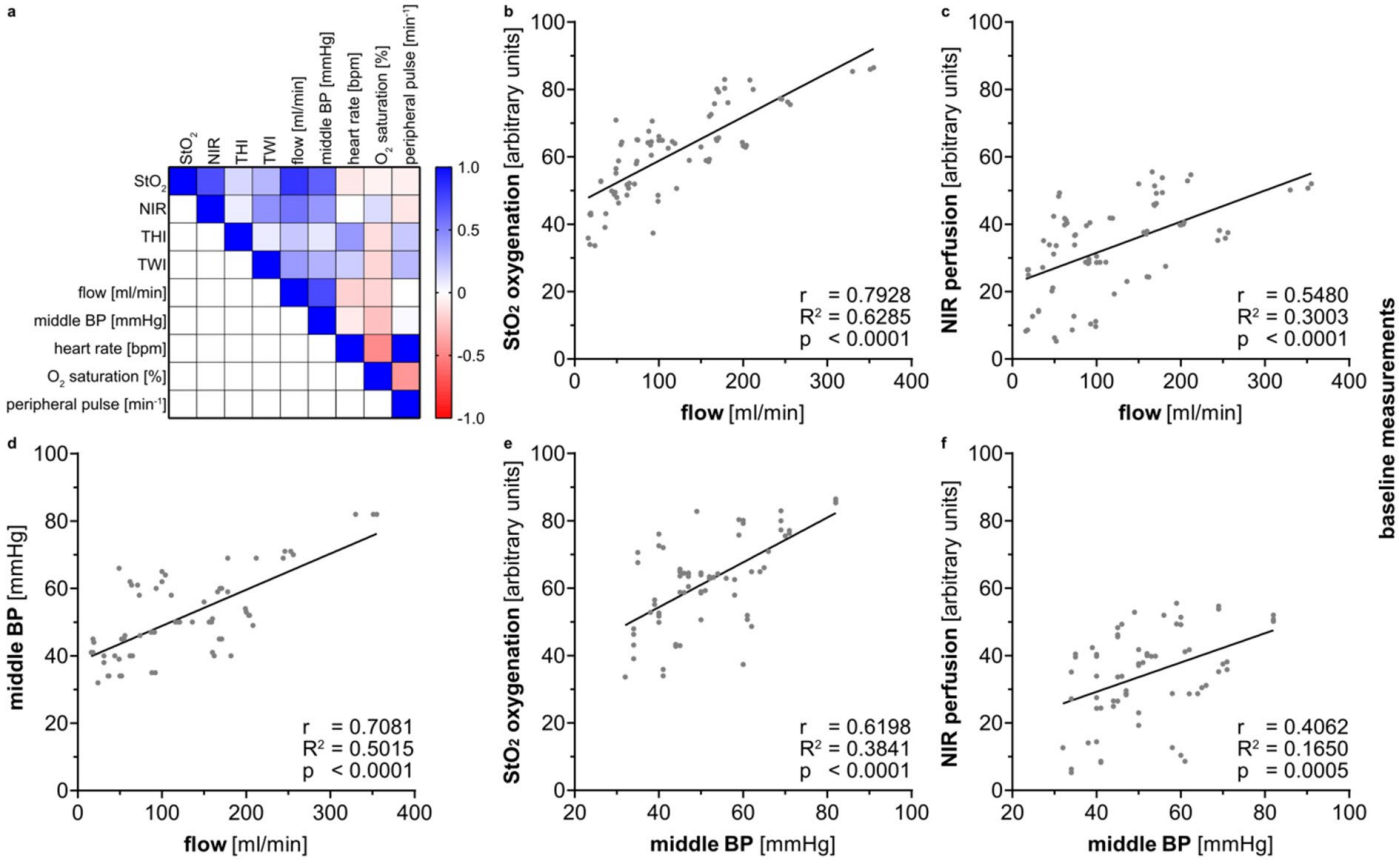
Correlation between flowmeter measurements, HSI index values and vital parameters in kidney measurements. (**a**) correlation matrix. (**b**) correlation between flow [ml/min] and HSI index values for StO_2_ (r = 0.7928; R^2^ = 0.6285). (**c**) correlation between flow [ml/min] and HSI index values for NIR (r = 0.5480; R^2^ = 0.3003). (**d**) correlation between flow [ml/min] and middle blood pressure [mmHg] (r = 0.7081; R^2^ = 0.5015). (**e**) correlation between middle blood pressure [mmHg] and HSI index values for StO_2_ (r = 0.6198; R^2^ = 0.3841). (**f**) correlation between middle blood pressure [mmHg] and HSI index values for NIR (r = 0.4062; R^2^ = 0.1650)**.**

HSI measurements in the animals were correlated with flowmeter and blood pressure measurements showing especially high correlation indices for HSI StO_2_ and flow (r = 0.79) as well as for HSI StO_2_ and mean arterial pressure (r = 0.62) (Fig. 8).

Independently, it could be shown that the application of the flowmeter probe itself attached to the renal artery does neither negatively affect the arterial inflow nor the reflectance spectrum and is not a confounder (Supplement Fig. 18).

## Discussion

Renal malperfusion is a situation regularly encountered or even required during visceral, transplant and urological surgical procedures. The amount of literature investigating ideal malperfusion concepts addressing clamping strategies, ischemia times and procedure sequences is staggering and growing.

While some research groups focus on the investigation of “selective versus hilar clamping”, reporting superior short-term renal function preservation for selective clamping strategies^16,17^, others entirely question the necessity for clamping and investigate “on-clamp versus off-clamp” differences during partial nephrectomy^3,18^, reporting a 7.3-fold increased risk of developing a severe chronic kidney disease in the on-clamp group. Yet, the focus is usually put on the outcome, while there is a lack of intraoperative visualization possibilities to provide objective measurements to correlate with that outcome. In routine cases of exclusively arterial manipulation this poses no challenge, as perfusion obstacles will have arterial origin. However, in cases of complex renal procedures such as full hilar clamping, intraoperative backtable perfusion with autotransplantation^19^ or conventional kidney transplantation, combined malperfusion situations can occur. Up to this day, there are no objective evaluation techniques for the differentiation of inflow or outflow obstruction and the surgeon is mainly depending on his or her experience and clinical judgement. The ramifications of compromised renal perfusion assessment reverberate beyond the immediate surgical context, resonating with the broader spectrum of patient care. A discerning evaluation not only mitigates the immediate risk of ischemic injury, but also predicates the long-term renal functional prognosis, delineating the trajectory of postoperative recovery. This underscores the imperative for a robust comprehension of renal perfusion intricacies and a growing commitment to the implementation of sophisticated methodologies. However, the three intraoperatively available technologies i.e. ultrasonic flowmetry, laser-Doppler flowmetry and ICG-fluorescence^5^ still have not shown clinical breakthroughs over recent years and represent mainly macrovascular or surrogate indicators.

Here, we present the first extensive spectral characterization of different intraoperative renal perfusion states based on an HSI kidney dataset across a total of 57 pigs and a dataset of 17 patients and suggest HSI in combination with ML as a promising technology for intraoperative kidney perfusion evaluation. The greatest advantage of HSI as opposed to ultrasonic flowmetry, which is currently the most widely established method, is seen in the microvascular approach and the capability to measure intraoperative tissue qualities other than exclusively arterial inflow with a spatial dimension to the renal parenchyma. These include oxygenation, hemoglobin and tissue water indices, but also extend even further when using HSI raw data and advanced computing with ML. The general advantage of HSI data is its high-dimensionality, which bears great potential for AI-based analysis methods as data complexity surpasses the ability of direct human comprehension. Only the combination of this complex and precise data together with a continuously growing size of systematic data collections and advanced AI-based analysis tools will enable long-term relevant results and identify objective information for diagnostic decisions.

In the past, the combination of ML with medical HSI has repeatedly proven feasible e.g. in microbiology rapidly classifying spectral signatures of bacterial colonies obtained from septic patients on blood agar plates and delivering real clinical benefit through advanced colony segmentation^20^. Also with other forms of electromagnetic imaging such as innovative biospeckle imaging, ML has proven to be well-suited e.g. for investigating wound healing^21^. However, the benefits of ML extend far beyond imaging as recent developments depict tremendous progress e.g. in the combination with robotics including the performance and versatility of soft robots in recent years^22^. Besides the availability of suitable real-world or synthetic training data, the design of ML methodology including network architecture, kernel size, number of epochs and learning rates becomes increasingly important^23^ in order to avoid the typical beginner mistakes of ML such as the overfitting pandemic in the mid-2000s.

However, when applying ML correctly following state of the art recommendations, the success stories can be limitless such as when Bu et al. developed a method to predict the prevalence risk of COVID-19 infection in dialysis patients with a prediction accuracy as high as 95.61%^24^.

Moreover, the beauty of newly developed ML algorithms and methods is that oftentimes these can be beneficially transferred to other applications. E.g. when Chi et al. developed a ML approach for automatic and objective gradation of wordwide terrorist attacks, this approach was also suitable for a broad range of gradation problems ranging from traffic accidents, meteorological and earthquake disasters to social behavior, and even urban planning^25^.

The clinical application of HSI in renal surgery has already been shown in multiple publications. Tetschke et al.^26^ first described HSI-based oxygen saturation monitoring during normothermic kidney perfusion, but only reported HSI index parameters to manually identify well-perfused and malperfused regions. No analysis of the underlying spectral reflectances, ML evaluations or correlations with clinical parameters were reported. Sommer et al.^27^ also described HSI-based evaluation during normothermic kidney perfusion in combination with ML to predict inulin clearance with impressive results. However, malperfusion was not differentiated and only investigated ex-vivo with limited evaluation of translation opportunity. Sucher et al.^7^ was the first to image kidneys in-vivo, but again only reported HSI index parameters with a very limited analytical approach. Ayala et al.^28^ first applied ML for automated analysis of kidney ischemia monitoring, but focused on real-time visualization using multispectral imaging severely limiting the spectral information content and only involved a low number of subjects.

Furthermore, the method was targeted to the specific use case of partial kidney resection and requires a personalized baseline measurement of well-perfused kidney. It therefore does not generalize to tissue perfusion classification in other procedures such as transplantation as ischemia detection was not based on spectral characteristics, but on out-of-distribution recognition. This need for calibration is hard to integrate into a clinical workflow and especially problematic in cases where there is no physiological baseline available such as during kidney transplantation. So, while the combination of HSI and renal malperfusion might not strike as a novelty per se, the systematic, methodical, analytical and translational depth and width of this study differentiating distinct types of malperfusion in-vivo and spectrally portraying temporal changes as well as gradual and partial malperfusion states in combination with state-of-the-art ML, the correlation with conventional flowmeter measurements and the comparison of animal and patient data is singular.

Limitations of the presented study include design elements of the ML application, the animal numbers and the naturally given limitations of an animal study.

Elaborating on the first limitation, the spectral reflectance of the “avascular” and the “arterial ischemia” group was highly congruent, indicating that in a situation of combined flow inhibition, the arterial inflow is the determining factor regarding spectral resemblance. ML-based differentiation between the two groups of “avascular” and “arterial ischemia” was found to be challenging taking the similarity of the descriptive spectral curves and PCA results (Figs. 1 and 2a) into consideration. For the ML approach, the baseline data from the 4 groups was therefore merged into 3 groups by combining these two entities. This acknowledges the fact that no proper clinical consequence would arise from this combination as in a situation of a combined perfusion problem, the arterial inflow would have to be addressed first and the component of venous congestion could then be identified and resolved in a second evaluation step.

As for the second limitation, the animal numbers are heterogenous for different groups and the numbers of human samples are rather small. This is due to the complexity of the procedures as well as the fact that physiological data from former experiments could be included in these analyses adding to the overall validity of the data^29^.

Thirdly, translating findings from animal experiments to human patients is a complex endeavor, with challenges rooted in the inherent differences in anatomy, physiology, and genetics between species. While we do have recorded human data, which provides some transferability insights, the limitations of animal models, ethical constraints, and the inability to fully replicate human disease heterogeneity still pose significant obstacles. Specifically, the animals in the present research project were young and otherwise healthy subjects as opposed to oncological or transplant patients who frequently present with significant comorbidities and therefore will have reduced collateral perfusion or tolerance towards gradually reduced perfusion. To bridge this translational gap effectively, a cautious, interdisciplinary approach is required, integrating animal data with human-centric research methods, such as clinical trials, to ensure the most informed and applicable therapeutic strategies.

Potential confounding factors of this study that could affect perfusion and spectral readings include surgical technique, anesthesia, and individual kidney physiology. Despite this list being not exhaustive, strong efforts were taken to minimize variability. The surgical technique was an in-house standard performed identically in all cases by the same surgeon. Renal clamping sites were always in the middle of the exposed renal artery and vein and clamping as well as reperfusion was recorded over a duration of 2 min each. An anesthesiologist monitored the animals throughout the procedure with standardized settings for ventilation and volume management. No hemorrhage occurred, no vasopressors were used and all individuals depicted normotensive values throughout. All animals were biologically as similar as possible with comparable weights and age from the same breeder with no signs of intraoperative adverse events.

The submitted manuscript provides the first extensive spectral characterization of kidney perfusion—physiological as well as malperfused—in a highly standardized and controlled setting. The core ideas and results of this study should not be mistakenly understood as for kidney alone, but—in its principles—show a unique type of data and diagnostic approach that can extend to a variety of other solid organs and change paradigm in future surgery.

Accurate intraoperative assessment of renal perfusion is a pivotal determinant during renal surgery. HSI results of this exploratory animal study suggest great potential to objectively describe, quantify and differentiate kidney malperfusion states as opposed to conventional surrogate techniques. Different degrees (narrowing vessel diameters) and types (arterial ischemia, venous congestion and combined malperfusion) of renal malperfusion show relevant differences in spectral reflectance as well as HSI index values such as HSI oxygenation with strictures becoming relevant at the transition of 2 mm to 1.5 mm in arterial vessel diameter and venous congestions being well-distinguishable from combined malperfusion or arterial ischemia using AI. The intricate interdependence of vascular dynamics and tissue oxygenation underscores the criticality of discerning even nuanced deviations from the physiological norm, an endeavor demanding the utmost precision and sophistication. ML algorithms seem feasible for intraoperative assistance systems for surgical questions regarding the evaluation of kidney perfusion. Results seem to transfer well to human data; however, clinical trials will be required to provide evidence to the claim of transferability to clinical scenarios and the aspiration of actual patient benefit.

## Methods

### Porcine model, anesthesia and monitoring

All experiments were granted with file reference number G-161/18 and G-262/19 by the Committee on Animal Experimentation of the Baden-Württemberg Regional Council in Karlsruhe, Germany. Experimental animals were managed as per institutional standard^30–34^ and according to German laws for animal use and care and according to the directives of the European Community Council (2010/63/EU) as well as the ARRIVE guidelines^35^. No protocol was registered due to the explorative, descriptive and hypothesis-generating nature of this study. An outbreeding pig strain (Sus scrofa domesticus) was used as the primary experimental animal model, deprived of food 24 h prior to surgery with free access to water. A total of 57 animals were used with a mean body weight of 36.8 (± 3.8) kg.

An intramuscular injection of azaperone (Stresnil 40 mg/ml by Elanco) with 6 mg/kg (≈ 6 ml = 240 mg) 15 min prior to further manipulation was conducted to achieve initial sedation. Next, an intramuscular injection of midazolam (Midazolam-hameln 5 mg/ml by hameln pharma plus gmbh) with 0.75 mg/kg (≈ 6 ml = 30 mg) and ketamine (Ketamin 10% by Heinrich Fromme) with 10 mg/kg (≈ 4 ml = 400 mg) was applied to reestablish analgosedation. Volume-controlled mode (Respirator: Primus Dräger Medical AG, Lübeck, Germany) with a tidal volume of 8 mL/kg, a positive-end-expiratory pressure of 5 mbar, an inspiration-to-expiration ratio (I:E ratio) of 1:2, and a fixed FiO_2_ of 0.5 was used for ventilation after endotracheal intubation with respiratory rate adjustments to reach an end-tidal CO_2_ of 40 ± 5 mmHg. Intraoperative anesthesia was achieved through balanced narcosis with 2 vol-% sevoflurane and the combination of i.v. 0.2 mg/kg/h midazolam (≈ 1.5 ml/h = 7.5 mg/h) and 8.75 mg/kg/h ketamine (≈ 3.5 ml/h = 350 mg/h) at a rate of 5 ml/h. No relaxant agents were applied. Peripheral oxygen saturation was monitored with a saturation probe on the tail. Every animal received two peripheral venous catheters into the ear veins and a central line in the jugular vein for i.v. application as well as an arterial line in the femoral artery for invasive blood measurement. Body temperature was sustained with electrical heat blankets. As part of the monitoring, invasive blood measurements and arterial inflow measurements in ml/min provided by an ultrasound flowmeter on the renal artery were recorded at the timepoints of HSI recordings.

The experiments were non-survival only; euthanasia was induced with intravenous potassium chloride and confirmed upon drop of end-expiratory CO_2_. There were no adverse events.

### Human data

Data from this porcine study was compared to a human dataset of patients undergoing kidney surgery recorded during the SPACE trial^15^. There were 88 recordings across 17 individuals for physiological human kidney and 16 recordings across 4 individuals for human kidney with accidental but certain arterial ischemia. All methods were carried out in accordance with relevant guidelines and regulations including the Declaration of Helsinki (DoH) and the World Medical Association's (WMA). All experimental protocols were approved by the Ethics Committee of the University of Heidelberg Faculty of Medicine with file reference number S-459/2020—SPACE Studie (SPectrAl Characterization of organs and tissuEs during surgery). Informed consent was obtained from all subjects or their legal guardian.

### Hyperspectral imaging

**Figure 9:**
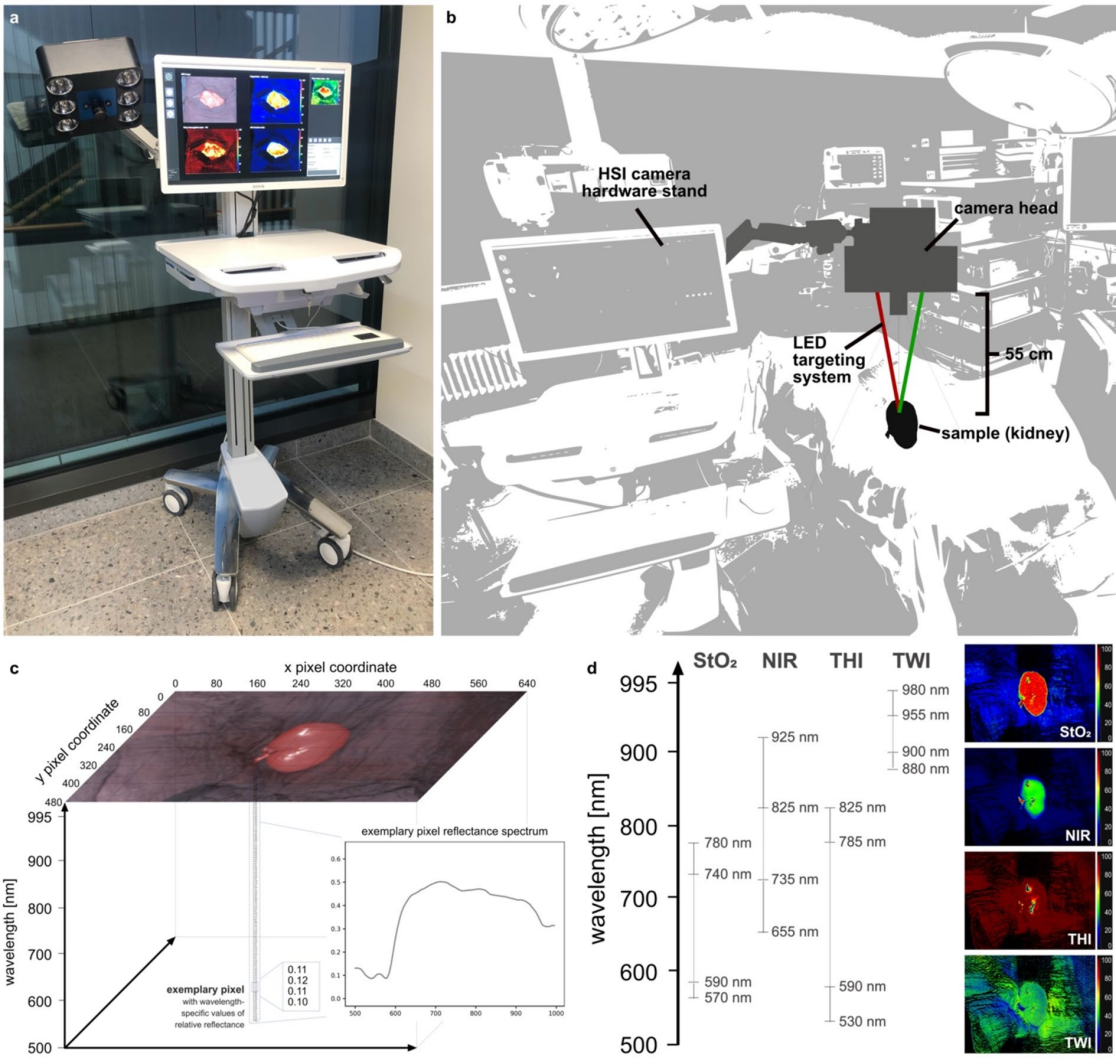
Technology and setup. (**a**) TIVITA Tissue from Diaspective Vision GmbH. (**b**) experimental setup with schematic depiction of the recording process. (**c**) structure of the three-dimensional data cube.** d**, relevant spectral bands for index image calculation.

HSI data of animals and patients was acquired using the TIVITA Tissue Halogen system from Diaspective Vision GmbH (Fig. 9). This system illuminates the respective field of view of around 20 × 27 cm with six integrated halogen lamps and provides a spectral resolution of 5 nm in the range from 500 to 995 nm for every recorded pixel.

Color-coded index pictures are calculated using the information of specific spectral bands as indicated in Fig. 9d and as obtained from the official formulas used by the HSI camera as cited in the literature^36^ in arbitrary percentage units. These include HSI Oxygenation Index (StO_2_), Near-Infrared-Perfusion Index (NIR), Tissue Hemoglobin Index (THI) and Tissue Water Index (TWI).

Mean and standard deviation (SD) reflectance spectra across animals were obtained by calculating the median spectrum over every pixel within the annotated region of interest (ROI) of one image and then the mean spectrum over every image within one pig. Primary data for all analyses was the original camera data after L1-normalization on pixel level.

### Surgical procedure and experimental groups in the animal model

**Figure 10:**
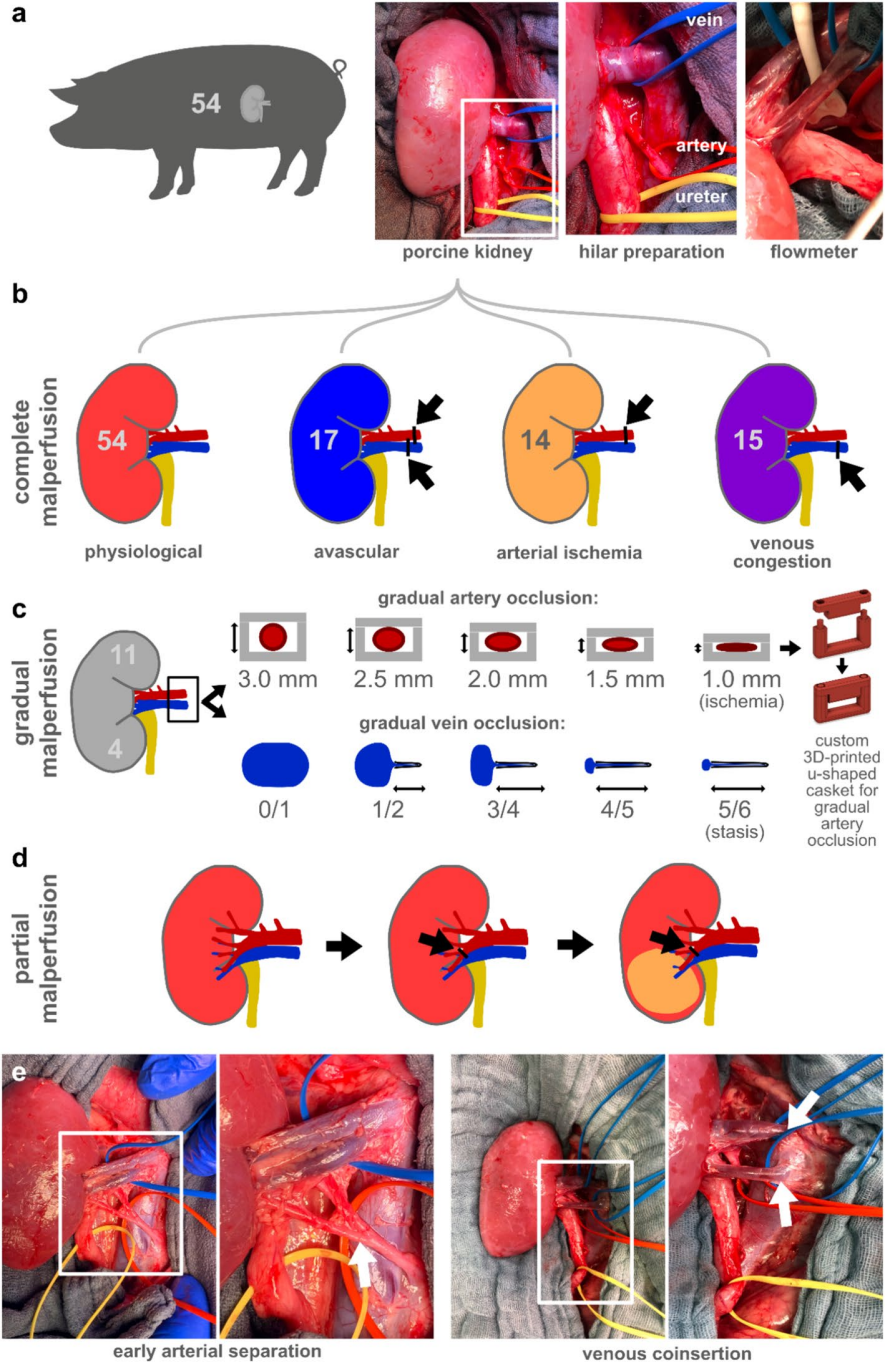
Investigational groups and recording protocol. (**a**) animal model after renal preparation and marking the hilar structures with surgical slings. (**b**) complete malperfusion. (**c**) gradual malperfusion model of artery and vein. (**d**) partial malperfusion by selective clamping of the inferior renal artery. (**e**) rare anatomical variants.

A total of 57 animals were used for the experiments. All data from all animals was used. The study was not hypothesis-driven, but exploratory. Consequently, there was no primary endpoint and no formal sample size calculation. All animal models received median laparotomy, bilateral renal mobilization with hilar preparation, marking the hilar structures with surgical slings. The experiments were divided into three investigational groups i.e. (1) complete malperfusion, (2) gradual malperfusion and (3) partial malperfusion (Fig. 10). For all experiments involving the renal artery, a flowmeter measuring flow in ml/min was placed around the artery for validation.

1. Complete malperfusion (malperfusion of the whole organ) was investigated comparing “physiological” perfusion with the 3 imaginable scenarios “avascular” kidney (combined inhibition of arterial inflow and venous outflow), “arterial ischemia” (selective inhibition of arterial inflow) and “venous congestion” (selective inhibition of venous outflow). Required malperfusion was reversibly induced by inhibiting either both simultaneously or arterial inflow or venous outflow only by placing vascular clamps on both vessels or the renal artery or the renal vein only as indicated in Fig. 10b. Extensive analyses on physiological organ data including kidney can be found in cited literature^29,37^.
2. Gradual malperfusion (gradual flow restriction of the whole organ) was investigated separately for gradual arterial ischemia and gradual venous stasis. For the induction of gradual arterial ischemia, custom 3D-printed polylactide u-shaped caskets with defined heights of 3 mm down to 1 mm in 0.5 mm steps were placed around the renal artery. A flowmeter probe confirmed that arterial inflow was not impaired in case of the 3 mm casket, was then adequately and comparably reduced for each following decreasing casket height and completely ceased with the 1 mm casket. For the induction of gradual venous congestion, the caskets could not be used due to the missing sturdiness of the venous wall structure. Instead, gradual venous congestion was induced by precisely placing vascular clamps on a defined fraction of the flattened vein diameter starting from completely open (0/1) all the way up to 5/6 of the diameter resulting in a clinically apparent massive venous congestion. All of these procedures were visualized in Fig. 10c.
3. Partial malperfusion (complete malperfusion of a part of the organ) was investigated by selectively clamping the inferior renal artery as indicated in Fig. 10d. This was always possible as with some minor preparation into the renal hilum, the separation of the renal artery into the superior and inferior renal artery could always be identified. However, investigating partial venous malperfusion using this model was not feasible as the renal vein did not regularly present anatomical separation in the hilar region outside the organ. Consequently, there was no possibility of selectively clamping an inferior renal vein.

### Image acquisition, annotation and processing in animals and patients

In order to prevent distortions of the measured reflectance spectra due to stray light, the tissue recordings were made while lights in the operating room were switched off and blinds closed. Images were recorded with a distance of 50 ± 5 cm between camera and organs perpendicularly to the organ surface. Annotations were created on the unprocessed HSI data using the HyperGUI (https://github.com/MIC-Surgery-Heidelberg/HyperGui). Non-semantic annotation was performed with a multi-point selection tool. Areas were selected as ROIs by omitting any areas with artifacts including tissue kinking, shade from the illumination, marginal areas, superficial blood vessels and fat, contamination with dyes or body fluids such as bile fluid, previous manipulation such as contusion or abrasion and possible impairment of perfusion such as thrombosis. The regions were selected with the aim of including only highly representative areas; therefore, it can be guaranteed that analyzed pixels were always 100% representative of the label. Consequently, there are additional adjacent pixels that could have been selected as well, but were not based on the judgment of the annotator and the premise to not include faulty or non-representative pixels under any circumstance. In case of several possible regions that were separated by aforementioned artifacts, the largest and most representative area was selected. For analyses and visualization, the spectral information was previously L1-normalized at pixel-level for increased uniformity.

### Vascular corrosion casting, digital volume tomography and scanning *electron* microscopy of animal organs

Vascular corrosion casting of the kidneys was obtained using Biodur E20 (Biodur Products, Heidelberg, Germany). After euthanasia, the abdominal aorta was cannulated, the distal thoracic aorta and iliac arteries were clamped and the inferior caval vein was incised. Perfusion was initiated by pressured infusion of 1,000 ml of Sterofundin (B. Braun) including 50,000 I.U. heparin through previously mentioned cannulation. Biodur E20 for injection was mixed at a ratio of 100:45 (v/v) Biodur E20 Plus and catalyst E20 and injected by manual pressure. The infusion was stopped after venous return of the casting agent was observed in the inferior caval vein and the material initially hardened for several minutes without further manipulation. The renal specimens were explanted and incubated for 12 h in a 40 °C water bath for further hardening. Tissue was removed with 15% (w/v) potassium hydroxide at room temperature over 3 days and the resulting vascular corrosion cast was subsequently rinsed in water. A digital vascular reconstruction was established performing DVT on the organ casts. Finally, scanning electron microscopy (SEM) samples were prepared from the parenchyma area. 20 mm × 10 mm samples of the outer layer were sputtered with 10 nm gold/platinum (80:20) (Leica EM ACE 600, Leica Microsystems GmbH, Wetzlar, Germany) and analyzed by SEM (Zeiss Leo Gemini 1530, Carl Zeiss AG, Oberkochen, Germany). SEM images were taken at different magnifications with an accelerating voltage of 2.0 kV (Supplement Fig. 17).

### Informatics and statistical analysis of animal and patient data

All code was developed and executed with PyCharm 2019.1.2 and Python 3.7. Data visualization and conventional statistical analyses were performed with GraphPad Prism 8.3.1. For all analyses, I indicates number of individuals while n indicates number of independent measurements. All statistical tests and p-values are purely descriptive due to the exploratory nature of the study and were performed on hierarchically aggregated data. A p-value < 0.05 is conventionally considered statistically significant; however, given the general criticism of p-values^38^, we encourage the readers to focus on the comprehensive presentation of raw values including transparently depicted boxplots and spectral curves that allow for a much more holistic view on the results. Yet, formal statistical testing for several groups was done with multiple comparison testing using ordinary one-way ANOVA for unpaired parametrically-distributed data and mixed-effects analysis with Geisser-Greenhouse correction for paired parametrically-distributed data with assumed equal standard deviations. Kruskal–Wallis test was used for unpaired data, while Friedman test was used for paired data. Analysis was assessor-blinded. Numerical values are provided with standard deviation in brackets. Significance levels were adjusted for multiple testing and were formally indicated with * for p ≤ 0.05, ** for p ≤ 0.01, *** for p ≤ 0.001, **** for p ≤ 0.0001 and n.s. for "not significant”. Graphs depict mean and standard deviation.

### Machine learning and Principal Component Analysis in animal data

For the ML approach, the baseline data from the 4 animal groups was merged into 3 groups by combining data from “avascular” and “arterial ischemia”. A ML architecture^39^ was used for kidney perfusion state classification of the median spectrum computed from the L1-normalized spectrum of all pixels in the annotated kidney regions. As a result, input to the ML architecture is a 100-dimensional vector and it outputs a class label indicating the predicted group. For training the ML architecture, the dataset is split into a training dataset consisting of 52 pigs (984 images) and a hold-out test dataset consisting of 5 pigs (202 images). The 5 pigs contained within the test dataset were selected such that all 3 groups were represented by each of the pigs. This criterion ensured that the test results were not confounded by pig-specific attributes. The test metrics were calculated using the hold-out test dataset, only after the ML pipeline was determined. For evaluating the performance of the ML architecture, balanced accuracy^40^ has been used. The ML architecture and hyperparameters have been kept consistent with the spectral organ fingerprint study^37^. Please refer to the methods section of the study for further details.

Lastly, a Principal Component Analysis (PCA)^41^ was applied on the preprocessed HSI data (L1-normalization of the spectrum on pixel level) and used to visualize spectral differentiability as described in previous publications^29^.

### Supplementary Information

Supplementary Information.

## Data Availability

The datasets used and analyzed during the current study are available from the corresponding author upon reasonable request.

## Abbreviations

AI: Artificial intelligence
CNN: Convolutional neural network
DVT: Digital volume tomography
HSI: Hyperspectral imaging
ML: Machine learning
PCA: Principal component analysis
ROI: Region of interest
SEM: Scanning electron microscopy
