# Supplementary material for: Spectral characterization of intraoperative renal perfusion using hyperspectral imaging and artificial intelligence
Source: Sci Rep. 2024 Jul 27;14:17262. doi: 10.1038/s41598-024-68280-3 (PMC11283474; doi:10.1038/s41598-024-68280-3)
Supplement: Supplementary file 1 — Supplementary Information. [file 41598_2024_68280_MOESM1_ESM.pdf]

Supplement

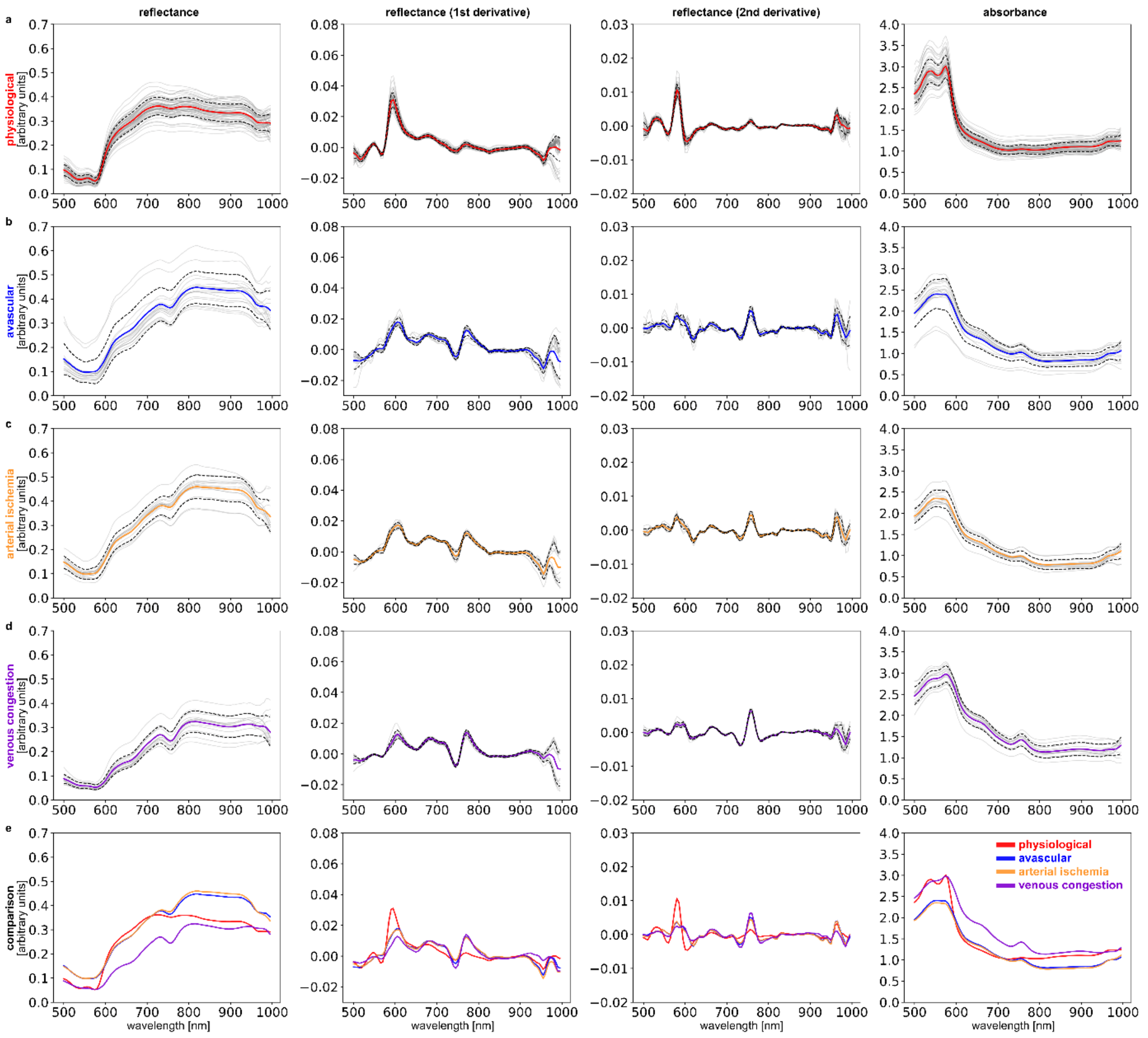

Supplement Figure 1 | Baseline of spectral kidney data for complete malperfusion in animals. Respective spectra for porcine kidney. **a**, physiological. **b**, avascular. **c**, arterial ischemia. **d**, venous congestion. **e**, overlay of group-specific spectra.

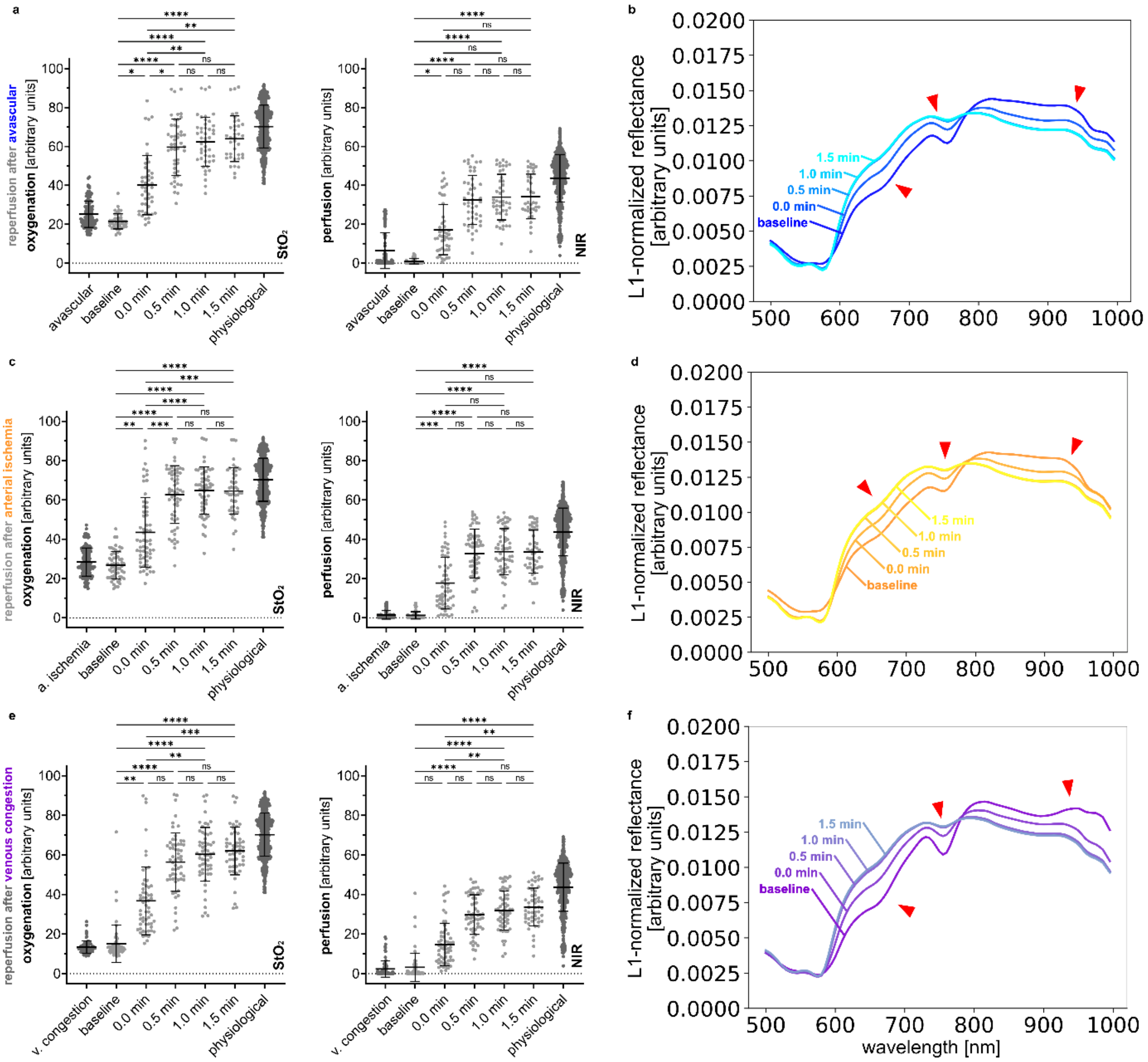

Supplement Figure 2 | Spectral changes over time in different states of kidney reperfusion after complete malperfusion in animals. Quantification of HSI index values (StO_2 and NIR) and overlay of multiple L1-normalized spectra according to changes over time. **a & b**, avascular. **c & d**, arterial ischemia. **e & f**, venous congestion.

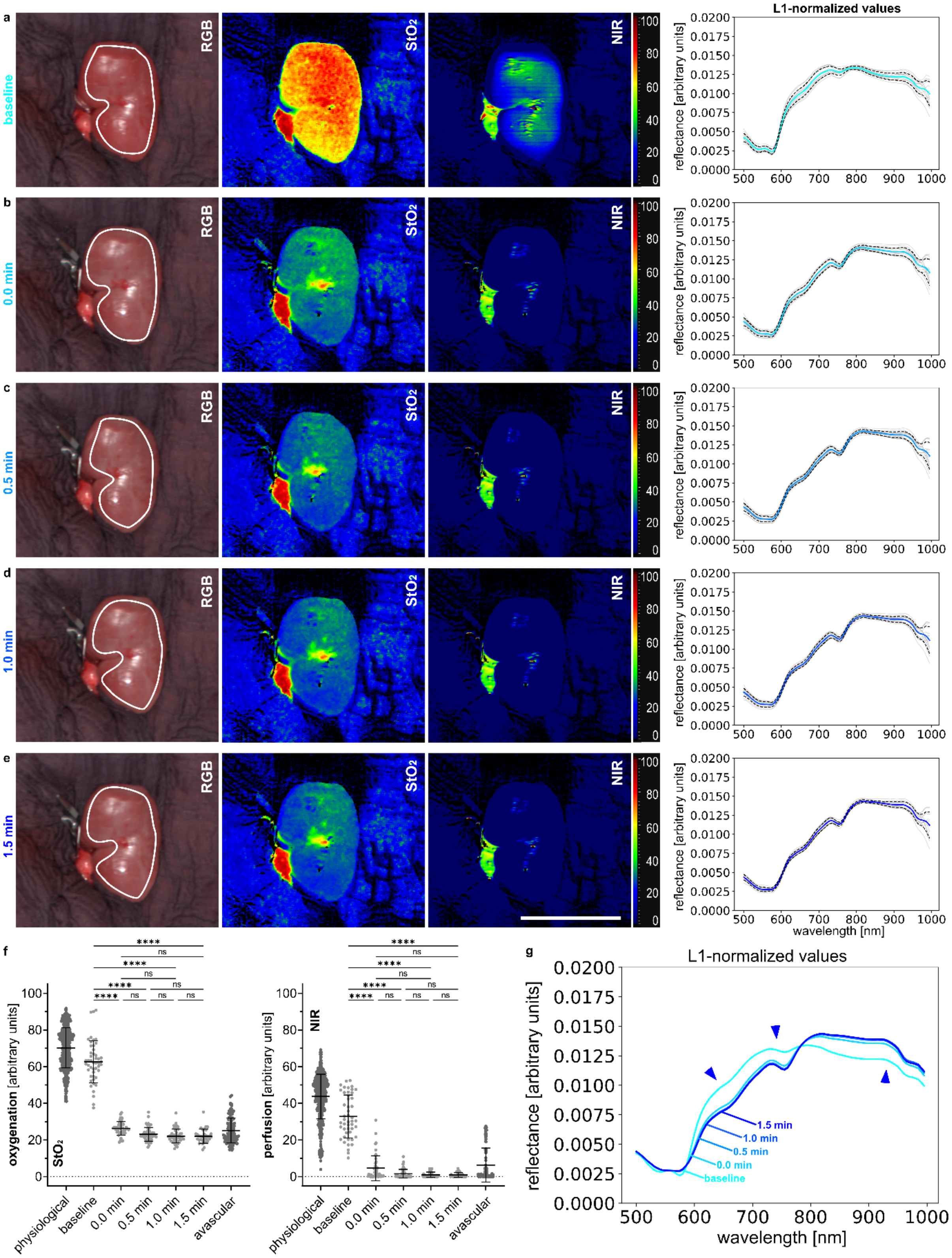

Supplement Figure 3 | Spectral changes over time of kidney avascularity. HSI color index pictures and respective spectra for porcine kidney. **a**, baseline corresponding to physiological kidney (I=14, n=48). **b**, kidney after 0 seconds of avascularity (I=14, n=48). **c**, kidney after 30 seconds of avascularity (I=14, n=49). **d**, kidney after 60 seconds of avascularity (I=14, n=48). **e**, kidney after 90 seconds of avascularity (I=13, n=32). **f**, quantification of HSI index values for StO_2 and NIR (process of avascularity).

g, overlay of multiple L1-normalized reflectances according to the changes over time. White scale bar equals 5 cm.

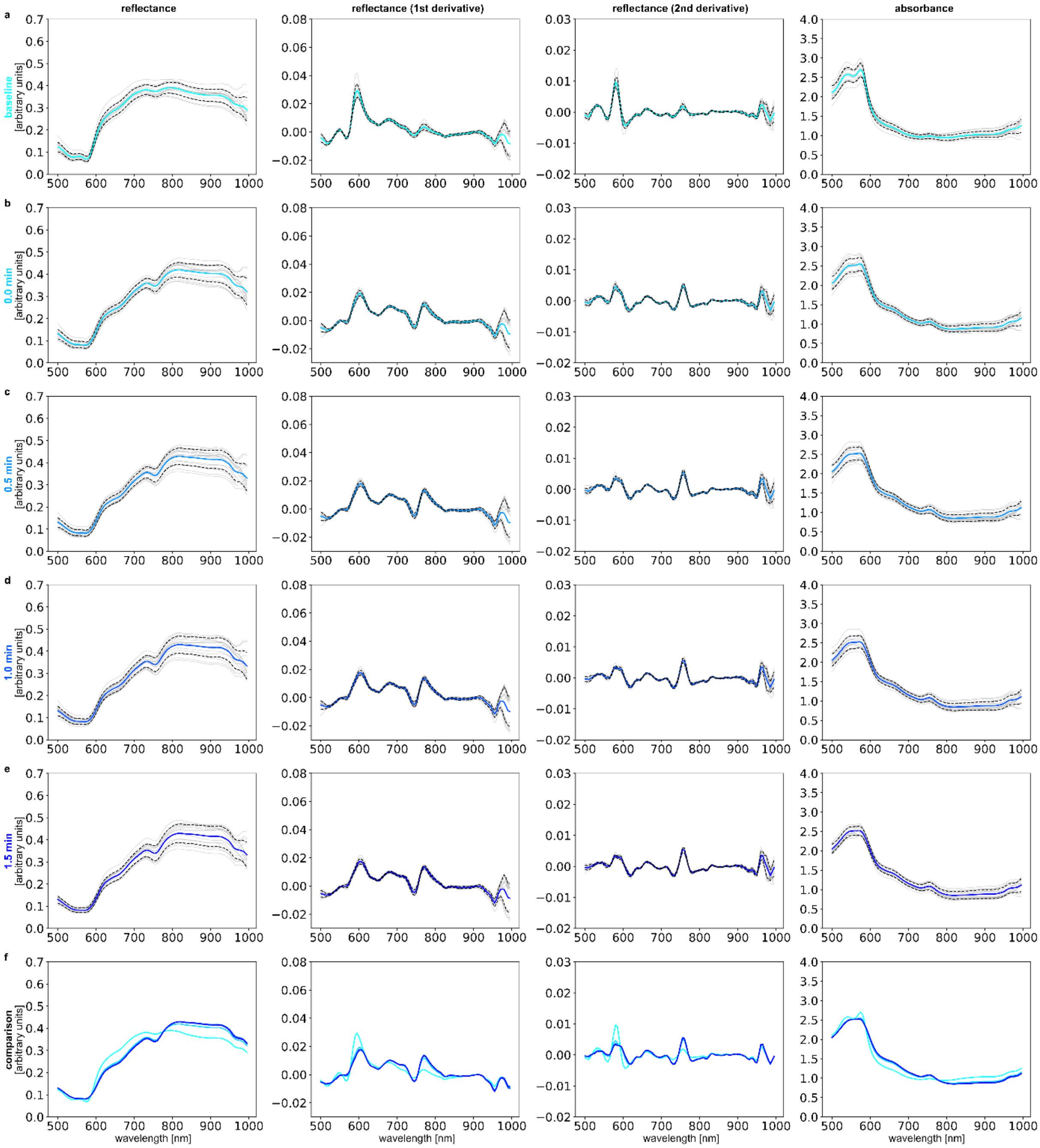

Supplement Figure 4 | Spectral changes over time of kidney avascularity. Respective spectra for porcine kidney. **a**, baseline corresponding to physiological kidney (I=14, n=48). **b**, kidney after 0 seconds of avascularity (I=14, n=48). **c**, kidney after 30 seconds of avascularity (I=14, n=49). **d**, kidney after 60 seconds of avascularity (I=14, n=48). **e**, kidney after 90 seconds of avascularity (I=13, n=32). **f**, overlay of group-specific spectra.

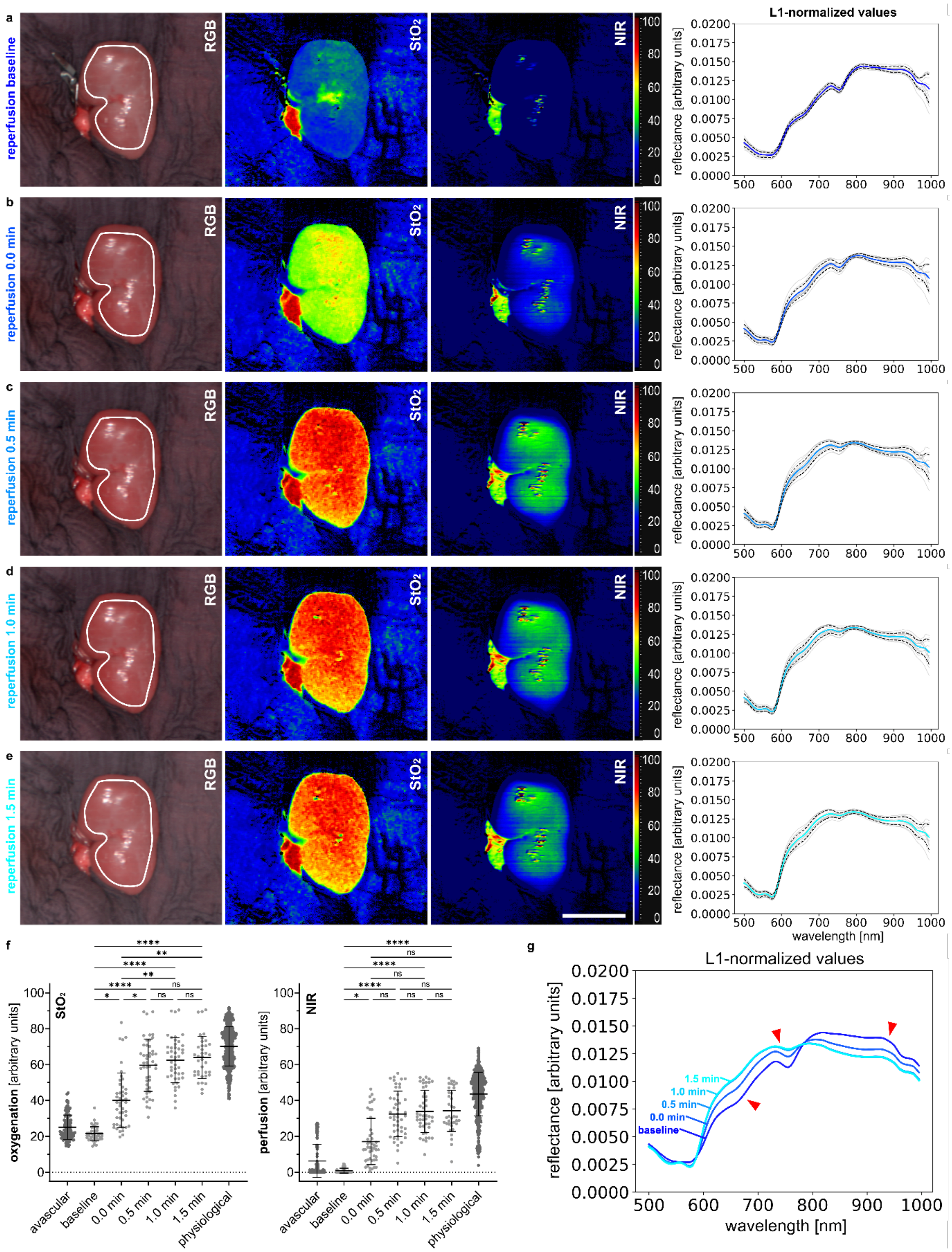

Supplement Figure 5 | Spectral changes over time of kidney reperfusion after avascularity. HSI color index pictures and respective spectra for porcine kidney. **a**, baseline corresponding to avascular kidney (I=13, n=46). **b**, kidney after 0 seconds of reperfusion (I=13, n=46). **c**, kidney after 30 seconds of reperfusion (I=13, n=47). **d**, kidney after 60 seconds of reperfusion (I=13, n=46). **e**, kidney after 90 seconds of reperfusion (I=12, n=37). **f**, quantification of HSI index values for StO_2 and NIR (process of

reperfusion after avascularity). **g**, overlay of multiple L1-normalized reflectances according to the changes over time. White scale bar equals 5 cm.

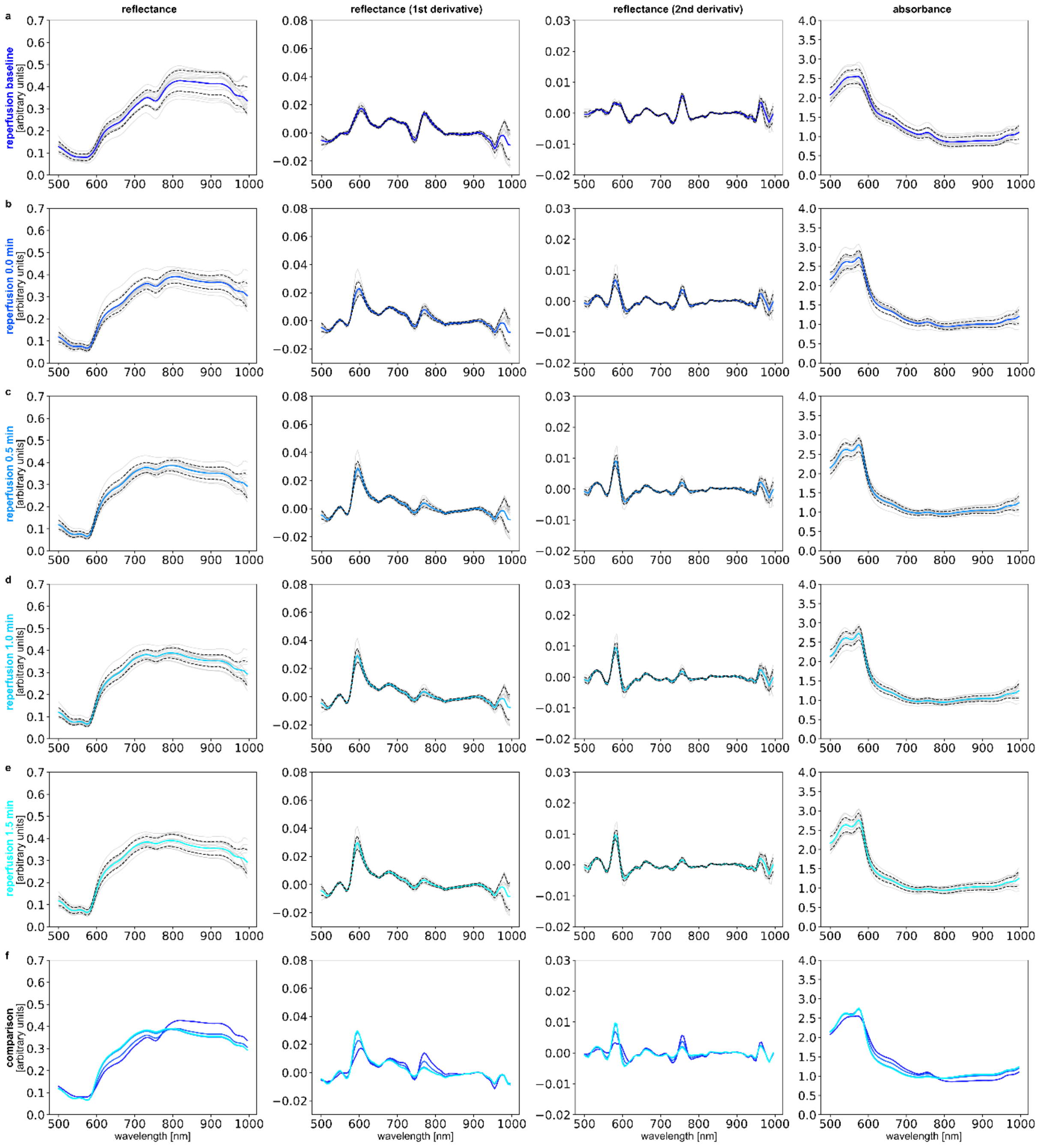

Supplement Figure 6 | Spectral changes over time of kidney reperfusion after avascularity. Respective spectra for porcine kidney. **a**, baseline corresponding to avascular kidney (l=13, n=46). **b**, kidney after 0 seconds of reperfusion (l=13, n=46). **c**, kidney after 30 seconds of reperfusion (l=13, n=47). **d**, kidney after 60 seconds of reperfusion (l=13, n=46). **e**, kidney after 90 seconds of reperfusion (l=12, n=37). **f**, overlay of group-specific spectra.

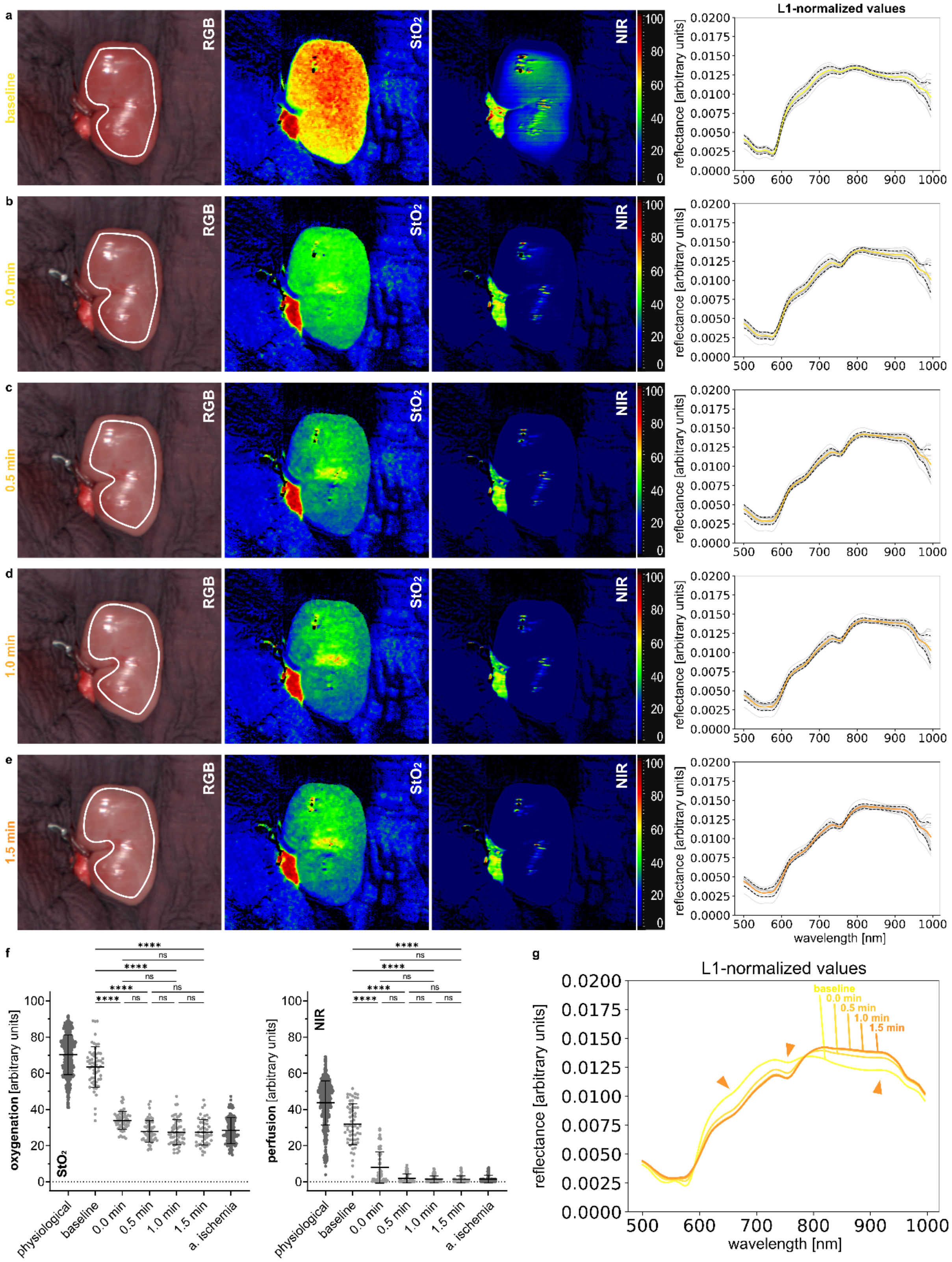

Supplement Figure 7 | Spectral changes over time of kidney arterial ischemia. HSI color index pictures and respective spectra for porcine kidney. **a**, baseline corresponding to physiological kidney (I=15, n=59). **b**, kidney after 0 seconds of arterial ischemia (I=15, n=59). **c**, kidney after 30 seconds of arterial ischemia (I=15, n=58). **d**, kidney after 60 seconds of arterial ischemia (I=15, n=59). **e**, kidney after 90 seconds of arterial ischemia (I=15, n=51). **f**, quantification of HSI index values for StO_2 and

NIR (process of ischemia). **g**, overlay of multiple L1-normalized reflectances according to the changes over time. White scale bar equals 5 cm.

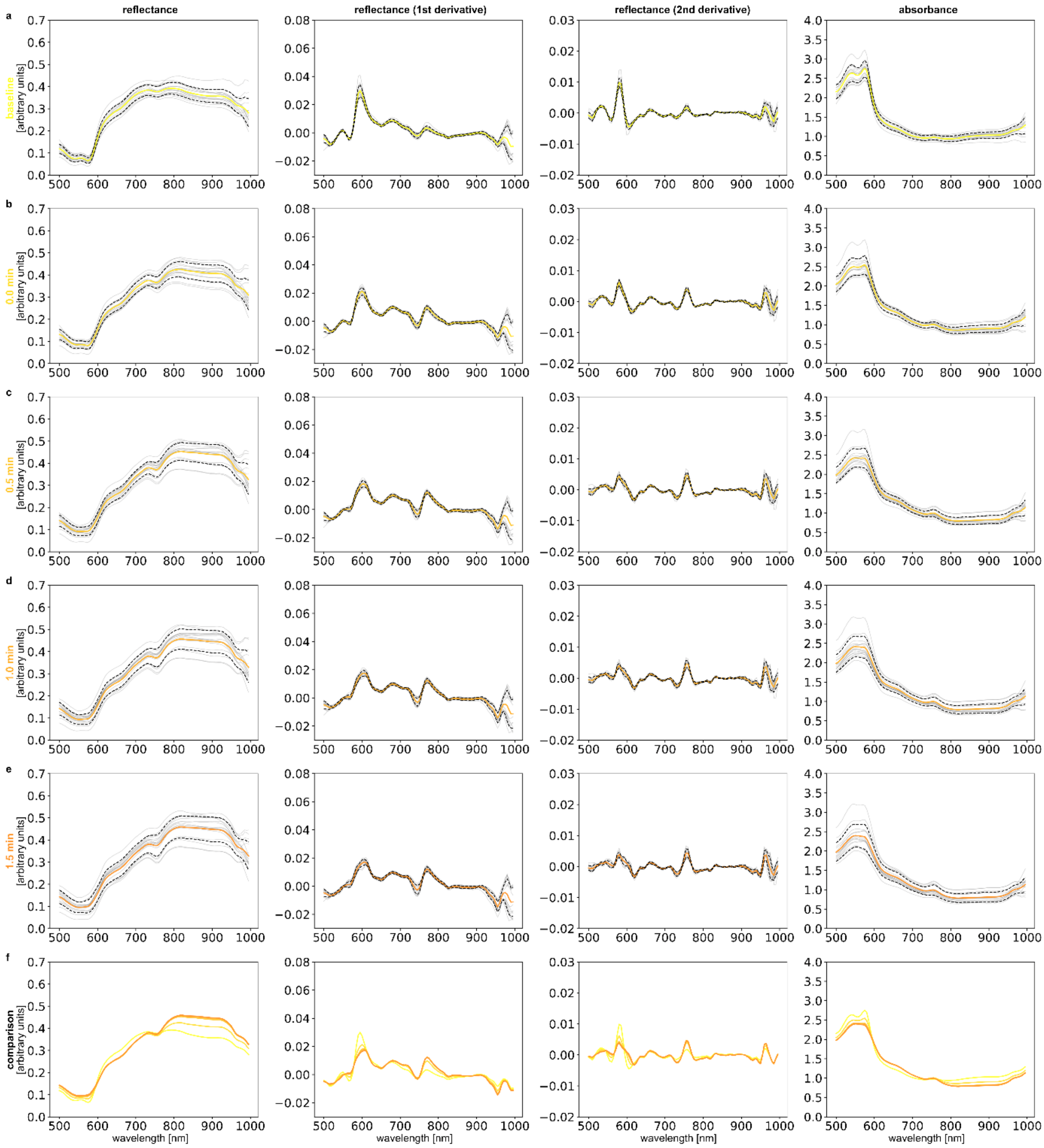

Supplement Figure 8 | Spectral changes over time of kidney arterial ischemia. RGB pictures and respective spectra for porcine kidney. **a**, baseline corresponding to physiological kidney (**l**=15, n=59). **b**, kidney after 0 seconds of arterial ischemia (**l**=15, n=59). **c**, kidney after 30 seconds of arterial ischemia (**l**=15, n=58). **d**, kidney after 60 seconds of arterial ischemia (**l**=15, n=59). **e**, kidney after 90 seconds of arterial ischemia (**l**=15, n=51). **f**, overlay of group-specific spectra.

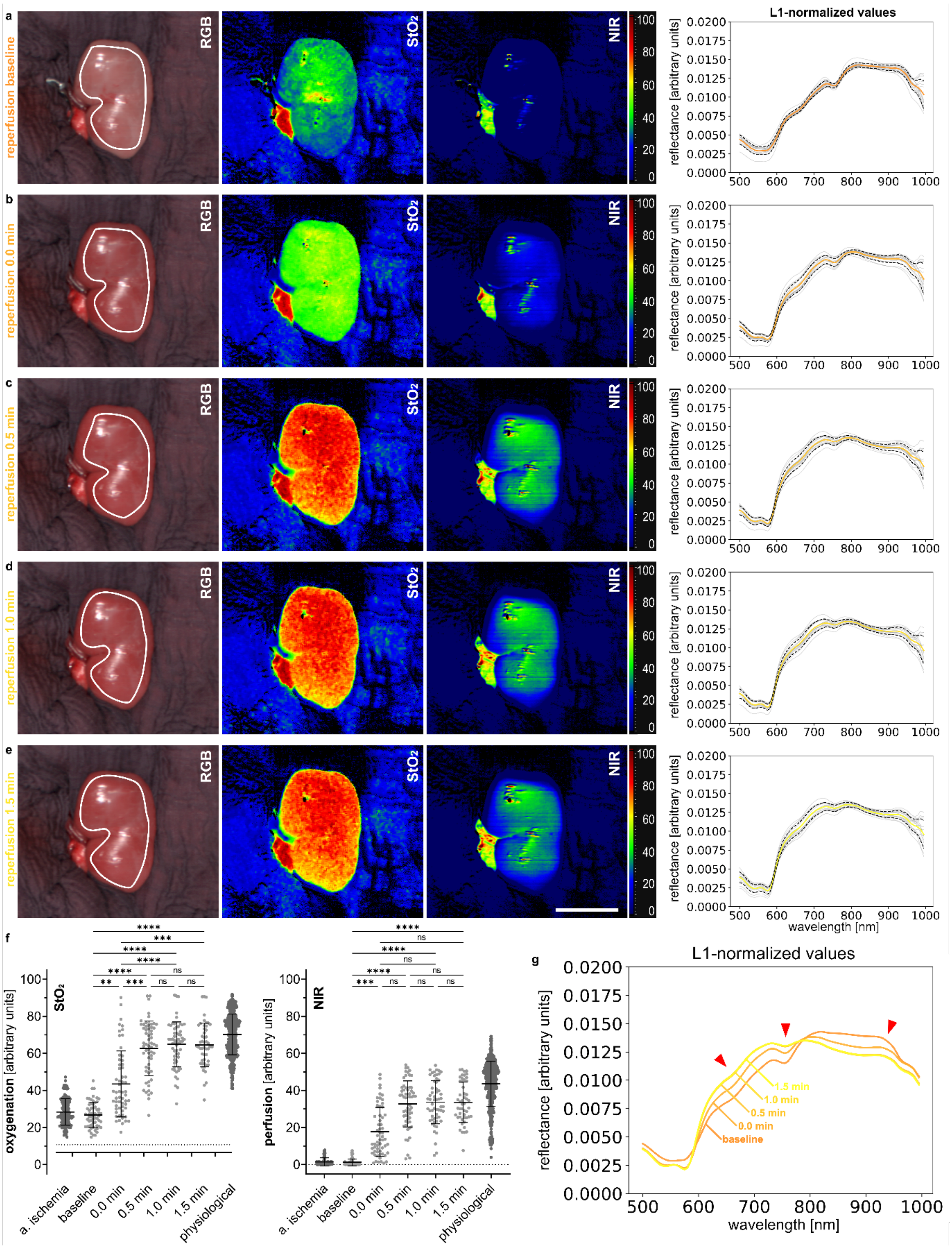

Supplement Figure 9 | Spectral changes over time of kidney reperfusion after arterial ischemia. HSI color index pictures and respective spectra for porcine kidney. **a**, baseline corresponding to ischemic kidney (I=15, n=59). **b**, kidney after 0 seconds of reperfusion (I=15, n=59). **c**, kidney after 30 seconds of reperfusion (I=15, n=59). **d**, kidney after 60 seconds of reperfusion (I= 15, n=58). **e**, kidney after 90 seconds of reperfusion (I=15, n=50). **f**, quantification of HSI index values for StO_2 and NIR

(process of reperfusion after arterial ischemia). **g**, overlay of multiple L1-normalized reflectances according to the changes over time. White scale bar equals 5 cm.

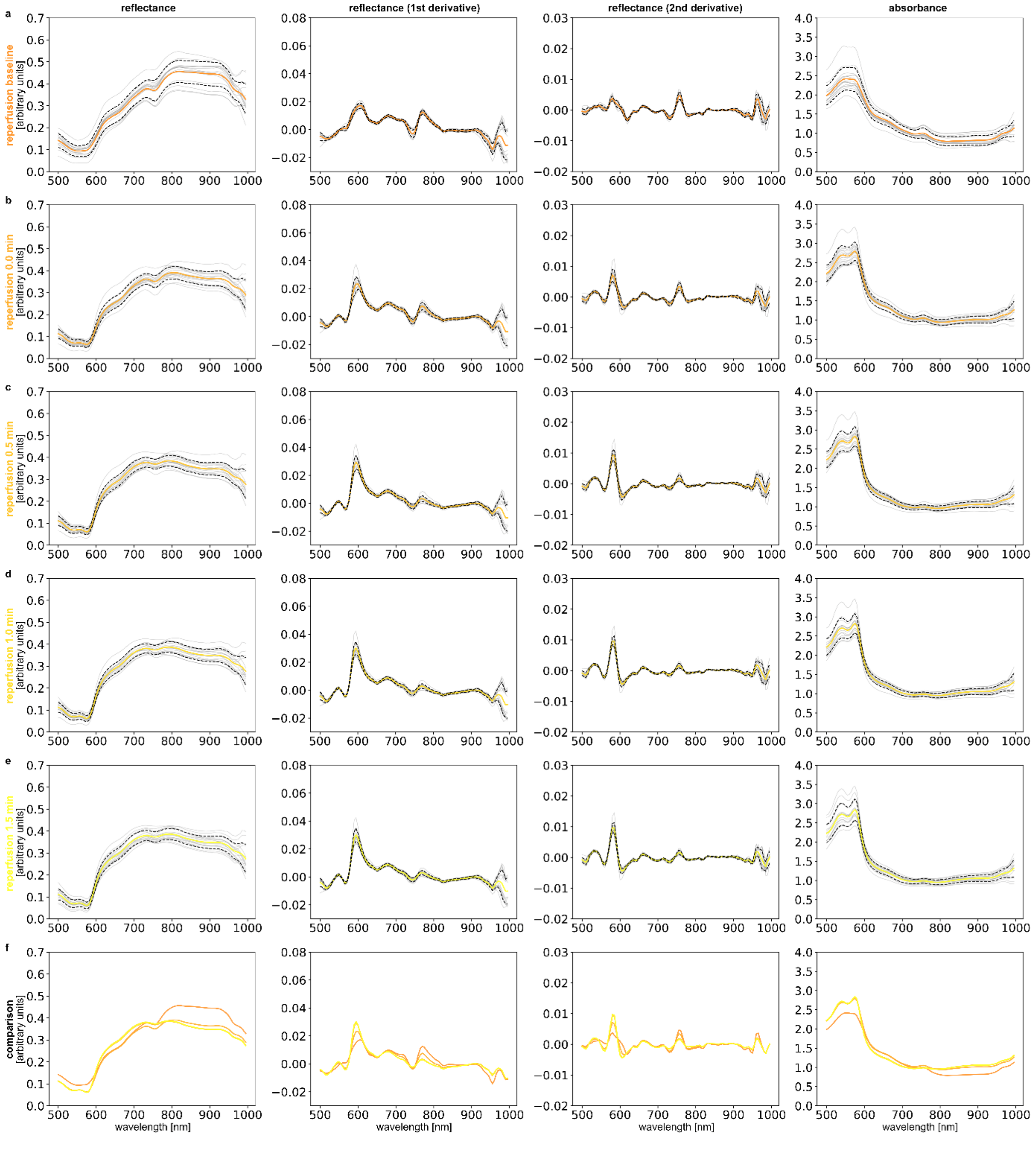

Supplement Figure 10 | Spectral changes over time of kidney reperfusion after arterial ischemia. Respective spectra for porcine kidney. **a**, baseline corresponding to ischemic kidney (I=15, n=59). **b**, kidney after 0 seconds of reperfusion (I=15, n=59). **c**, kidney after 30 seconds of reperfusion (I=15, n=59). **d**, kidney after 60 seconds of reperfusion (I=15, n=58). **e**, kidney after 90 seconds of reperfusion (I=15, n=50). **f**, overlay of group-specific spectra.

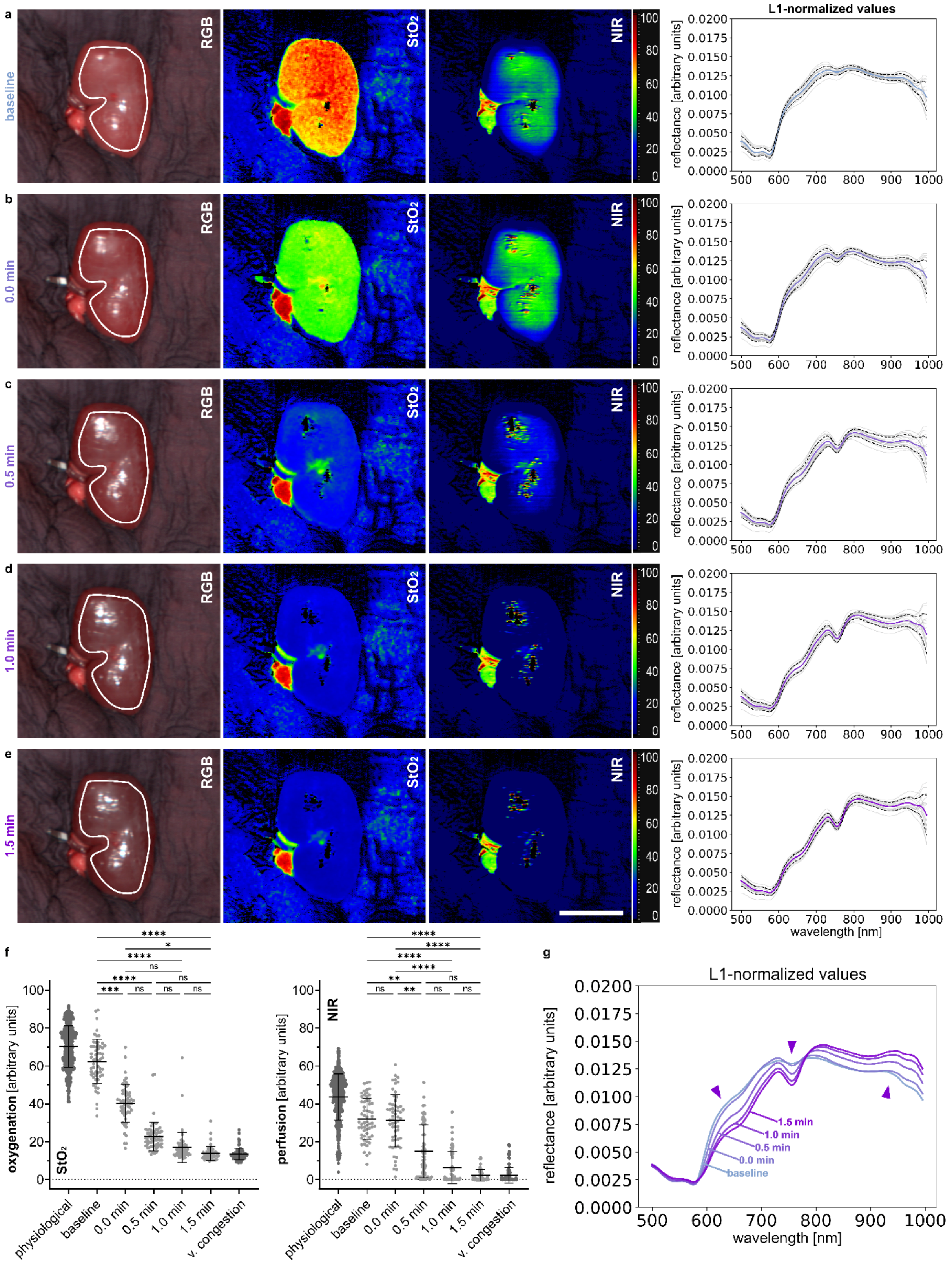

Supplement Figure 11 | Spectral changes over time of kidney stasis. HSI color index pictures and respective spectra for porcine kidney. **a**, baseline corresponding to physiological kidney (l=15, n=58). **b**, kidney after 0 seconds of venous congestion (l=15, n=58). **c**, kidney after 30 seconds of venous congestion (l=15, n=59). **d**, kidney after 60 seconds of venous congestion (l=15, n=59). **e**, kidney after 90 seconds of venous congestion (l=15, n=49). **f**, quantification of HSI index values for StO_2 and NIR

(process of stasis). **g**, overlay of multiple L1-normalized reflectances according to the changes over time. Scale bar equals 5 cm.

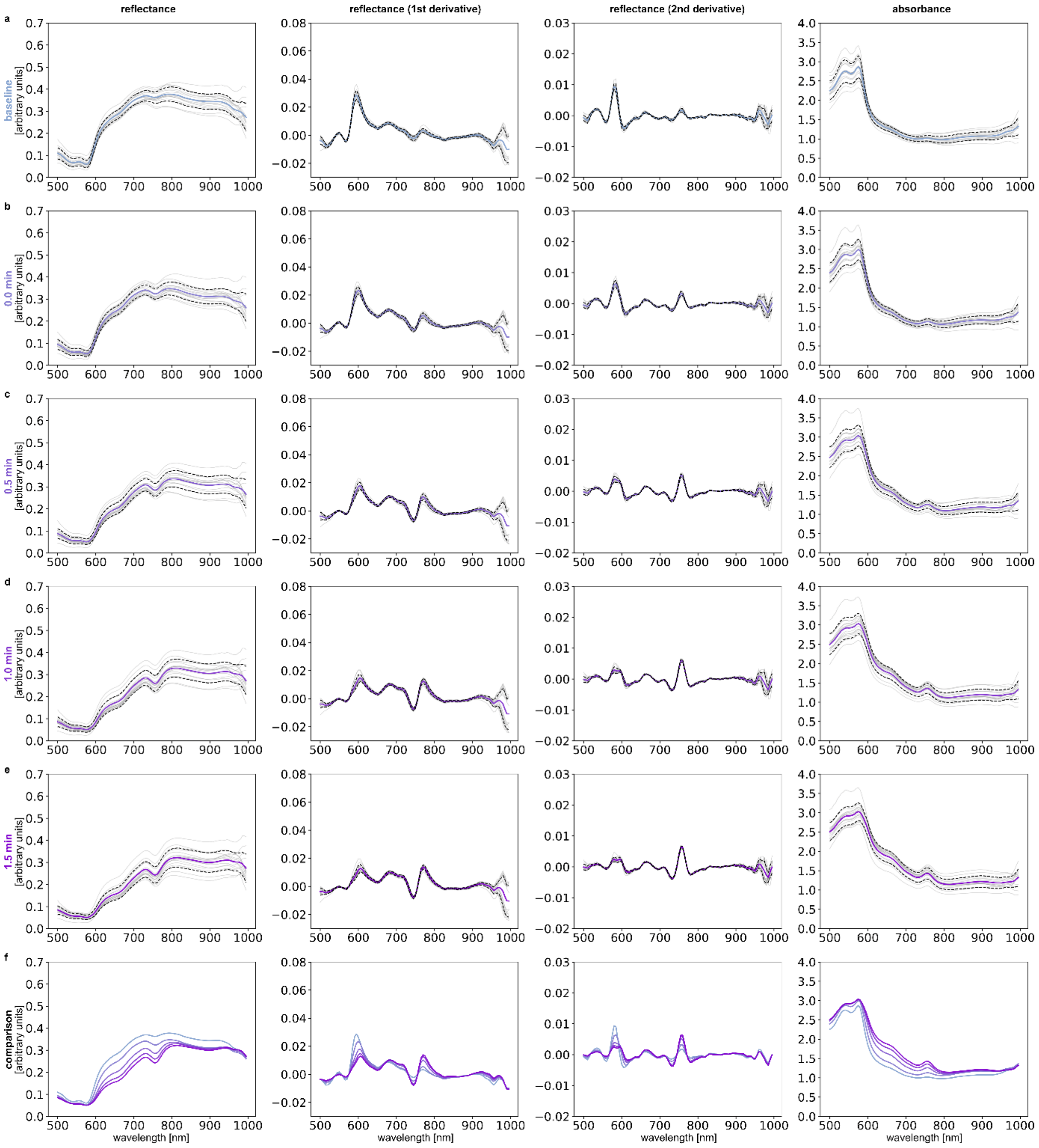

Supplement Figure 12 | Spectral changes over time of kidney stasis. Respective spectra for porcine kidney. **a**, baseline corresponding to physiological kidney (**I**=15, n=58). **b**, kidney after 0 seconds of venous congestion (**I**=15, n=58). **c**, kidney after 30 seconds of venous congestion (**I**=15, n=59). **d**, kidney after 60 seconds of venous congestion (**I**=15, n=59). **e**, kidney after 90 seconds of venous congestion (**I**=15, n=49). **f**, overlay of group-specific spectra.

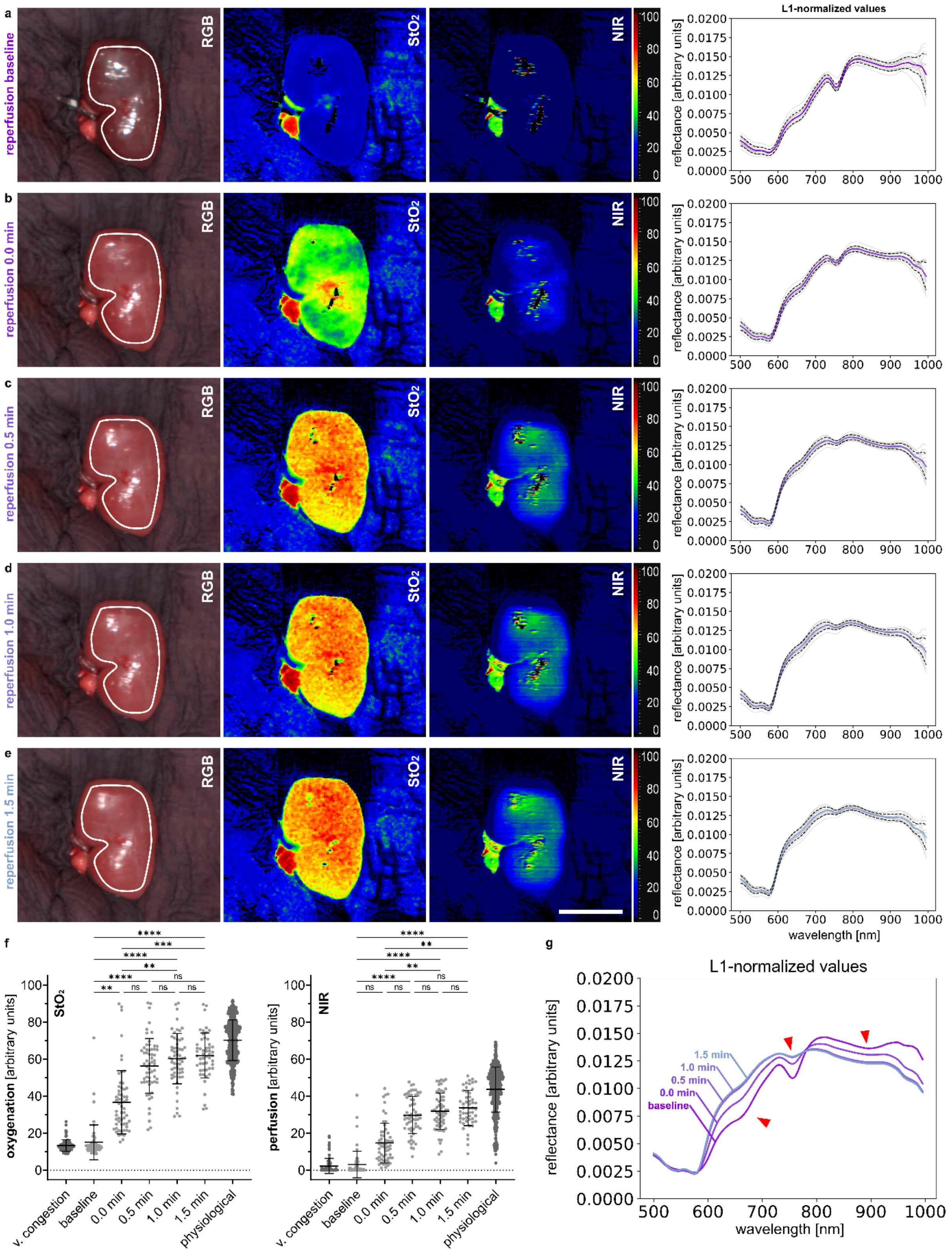

Supplement Figure 13 | Spectral changes over time of kidney reperfusion after venous congestion. HSI color index pictures and respective spectra for porcine kidney. **a**, baseline corresponding to congested kidney (l=15, n=59). **b**, kidney after 0 seconds of reperfusion (l=15, n=59). **c**, kidney after 30 seconds of reperfusion (l=15, n=60). **d**, kidney after 60 seconds of reperfusion (l=15, n=59). **e**, kidney after 90 seconds of reperfusion (l=15, n=52). **f**, quantification of

HSI index values for StO_2 and NIR (process of reperfusion after venous congestion). **g**, overlay of multiple L1-normalized reflectances according to the changes over time. White scale bar equals 5 cm.

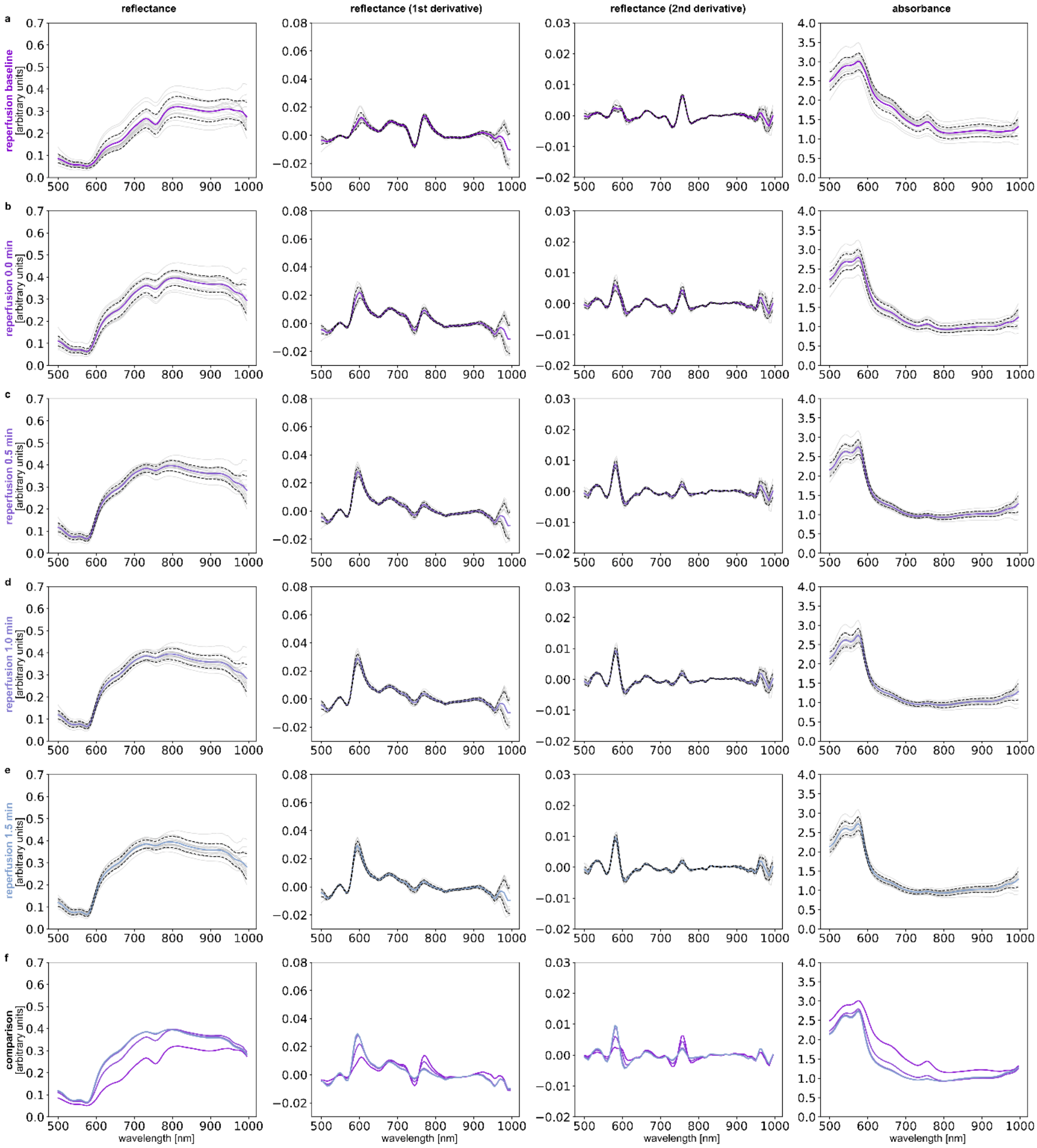

Supplement Figure 14 | Spectral changes over time of kidney reperfusion after venous congestion. Respective spectra for porcine kidney. **a**, baseline corresponding to congested kidney (**I**=15, n=59). **b**, kidney after 0 seconds of reperfusion (**I**=15, n=59). **c**, kidney after 30 seconds of reperfusion (**I**=15, n=60). **d**, kidney after 60 seconds of reperfusion (**I**=15, n=59). **e**, kidney after 90 seconds of reperfusion (**I**=15, n=52). **f**, overlay of group-specific spectra.

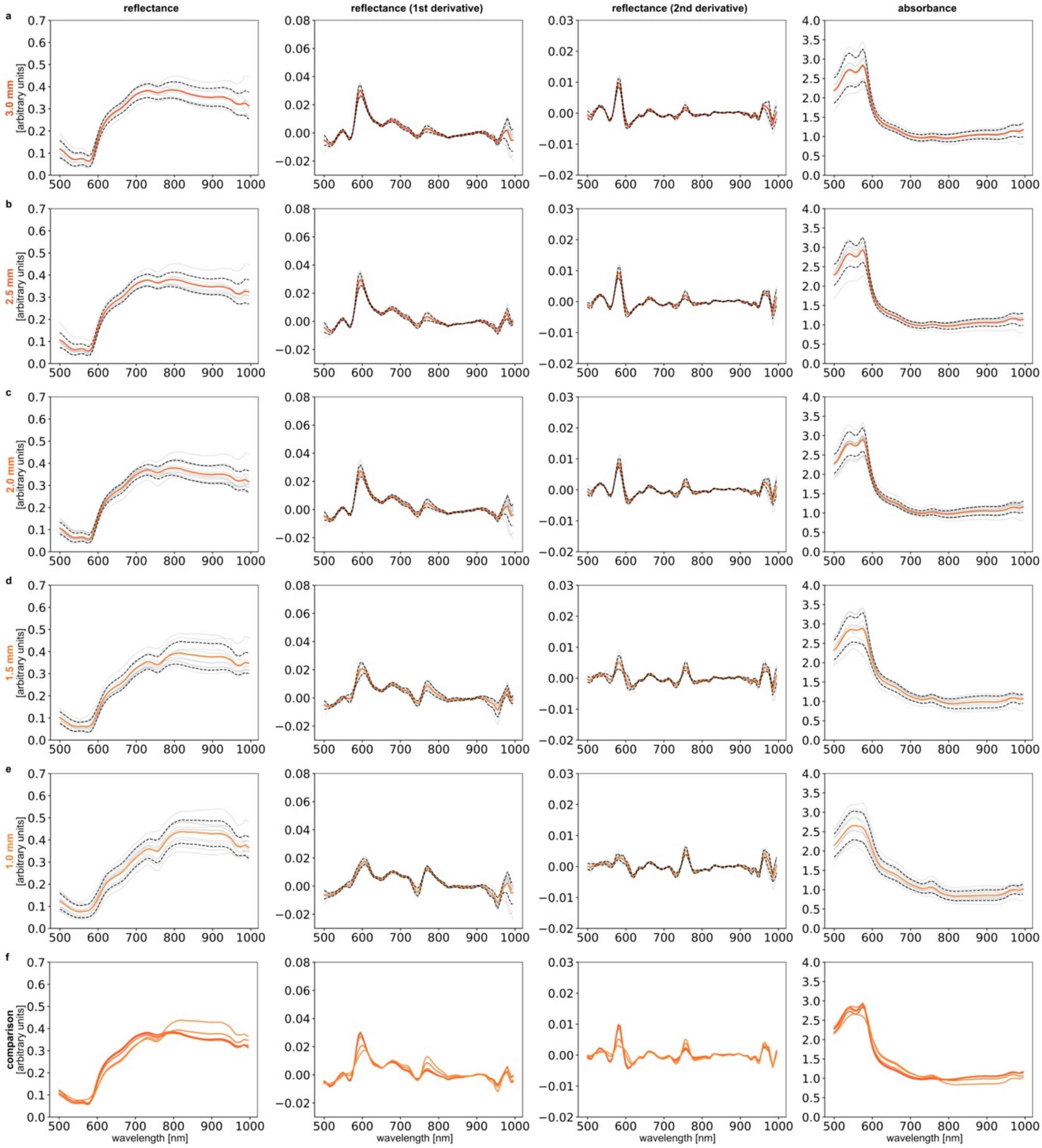

Supplement Figure 15 | Gradual clamping of the renal arteries with various gaps. Respective spectra for porcine kidney. **a**, gradually ischemic kidney with a 3.0 mm gap on the artery (**I**=8, n=12). **b**, gradually ischemic kidney with a 2.5 mm gap on the artery (**I**=8, n=13). **c**, gradually ischemic kidney with a 2.0 mm gap on the artery (**I**=11, n=21). **d**, gradually ischemic kidney with a 1.5 mm gap on the artery (**I**=9, n=19). **e**, gradually ischemic kidney with a 1.0 mm gap on the artery (**I**=11, n=18). **f**, overlay of group-specific spectra.

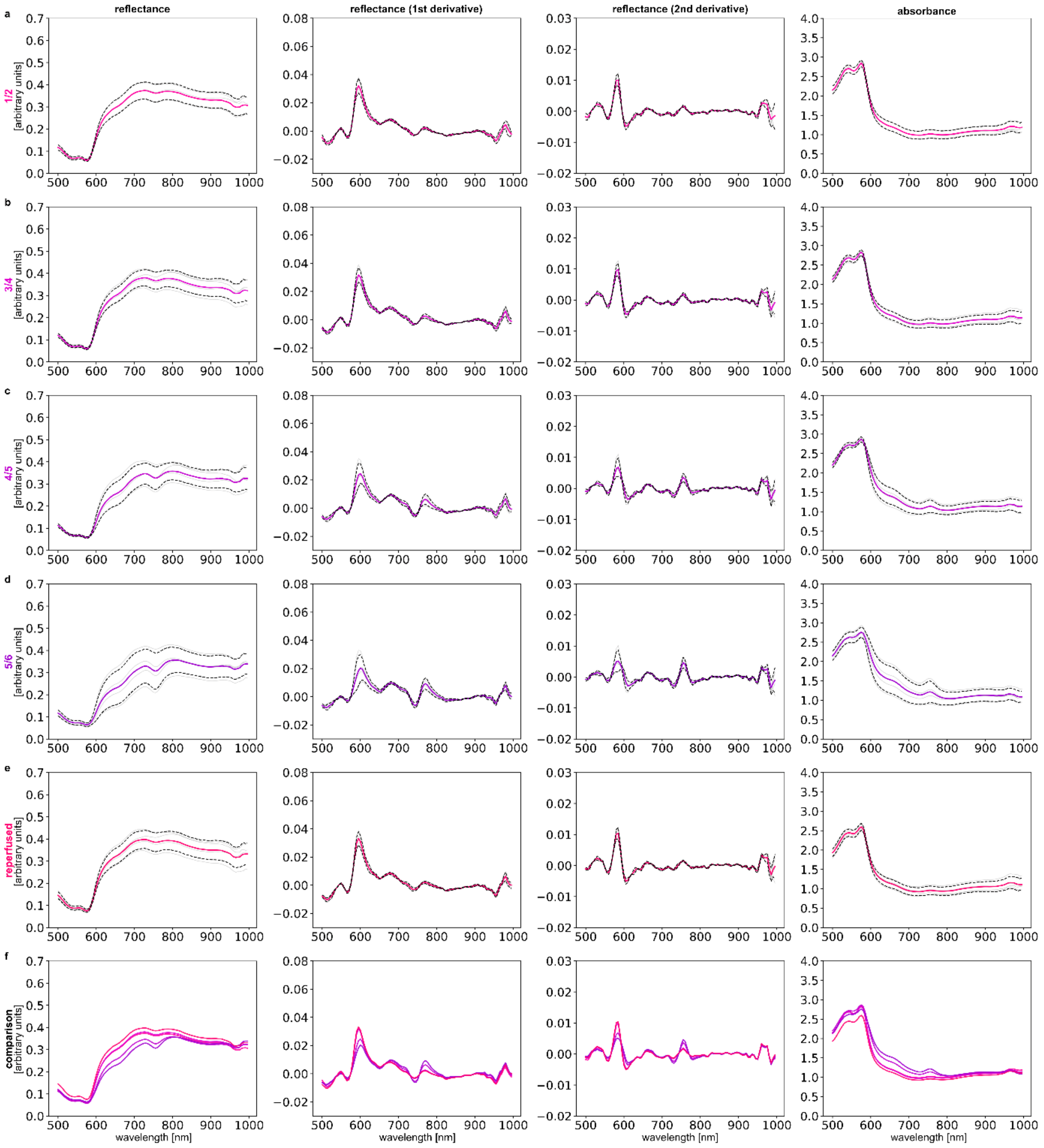

Supplement Figure 16 | Gradual clamping of the renal veins with different steps of occlusion. Respective spectra for porcine kidney. **a**, kidney with half occluded vein (l=3, n=6). **b**, kidney with three-quarters occluded vein (l=4, n=11). **c**, kidney with four-fiths occluded vein (l=4, n=12). **d**, kidney with five-sixths occluded vein (l=4, n=8). **e**, completely reperfused kidney after the process of gradual stasis (l=4, n=13). **f**, overlay of group-specific spectra.

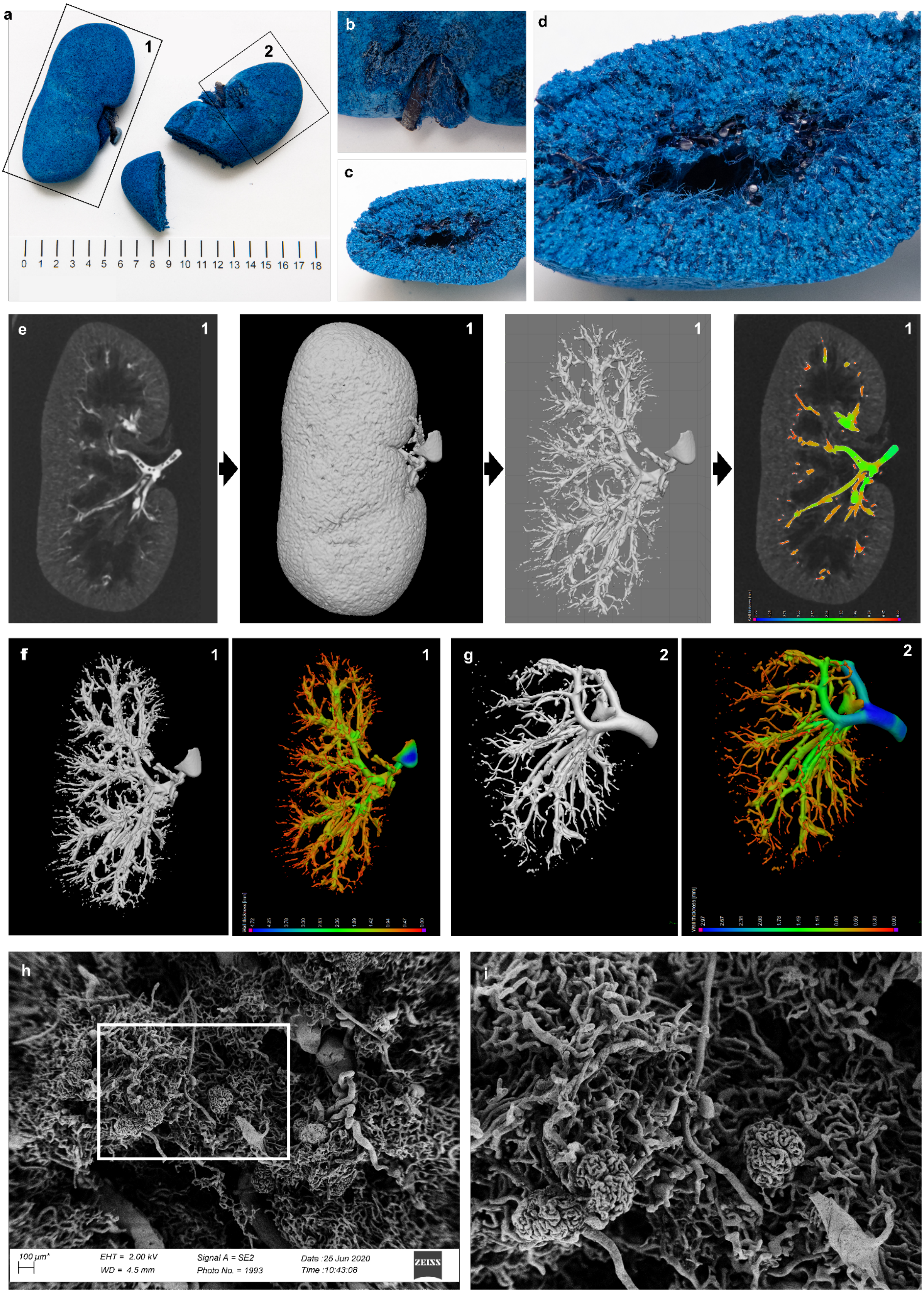

Supplement Figure 17 | Vascular Corrosion Casting. **a**, Vascular corrosion casting of left (1) and right (2) porcine kidney. **b**, renal hilum. **c**, renal parenchyma. **d**, magnification of the renal parenchyma. **e**, procedure of vascular reconstruction through digital volume tomography of renal

vascular corrosion casting. **f**, vascular reconstruction of the whole kidney. **g**, vascular reconstruction of inferior renal artery perfusome. **h**, scanning electron microscopy of renal vascular corrosion casting. **i**, magnification of visible glomeruli.

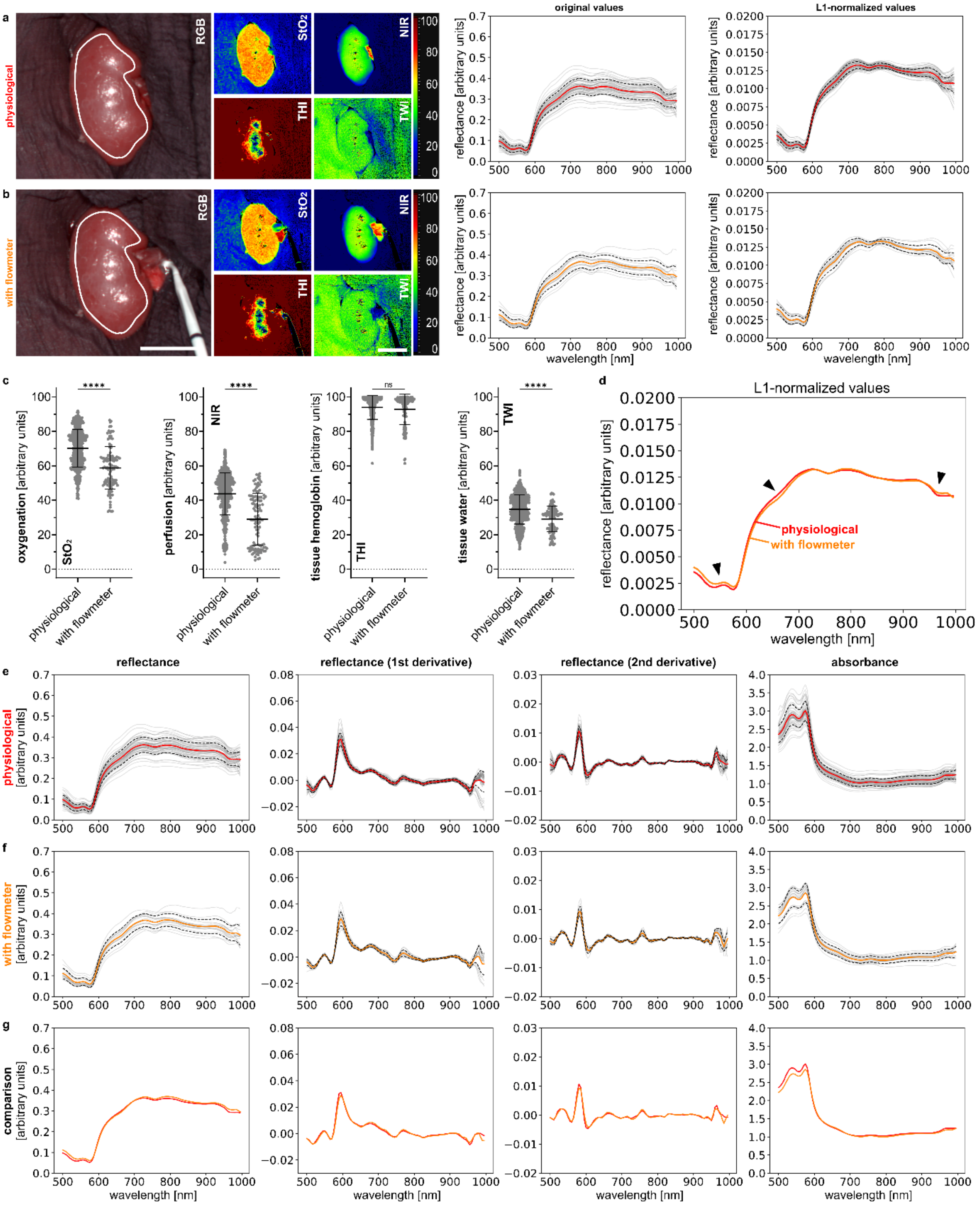

Supplement Figure 18 | Effect of Flowmeter on Renal Spectral Changes. HSI color index pictures and respective spectra for porcine kidney. **a**, kidney without flowmeter (I=54, n=757). **b**, kidney with flowmeter (I=16, n=115). **c**, quantification of HSI index values for StO_2, NIR, THI and TWI. **d**, overlay

of multiple L1-normalized reflectances. **e-g**, extensive comparison of respective spectra for porcine kidney without and with flowmeter on renal artery. Scale bar equals 5 cm.
